# Process-Modulated Flavor Formation and Establishment of Predictive Modeling for Aroma in Spicy Anchovies

**DOI:** 10.3390/molecules31010057

**Published:** 2025-12-23

**Authors:** Zishan Liao, Qian Liu, Wenli Kang, Tao Feng, Zemin Ding, Shixian Yin, Shiqing Song

**Affiliations:** 1 Faculty of Flavour Fragrance and Cosmetics, Shanghai Institute of Technology, Shanghai 201418, China; 19121696503@163.com (Z.L.); liuqian@sit.edu.cn (Q.L.); fengtao@sit.edu.cn (T.F.); 2 Jinzai Food Group Co., Ltd., Yueyang 410400, China; kangwenli2012@163.com (W.K.); dzm0929@163.com (Z.D.); yinshixian@jinzaifood.com.cn (S.Y.); 3 Pingjiang Jinzai Food Co., Ltd., Yueyang 410400, China

**Keywords:** spicy anchovies, flavor wheel, gas chromatography-mass spectrometry/olfactometry, partial least squares regression analysis, prediction model

## Abstract

Research on spicy anchovies lacks dedicated sensory frameworks, reliable aroma identification, and systematic processing–flavor insights. In this study, 21 spicy anchovy samples with different processing parameters were selected as research objects. The effects of process modifications on the sensory attributes and aroma composition of spicy anchovies were investigated through sensory evaluation and aroma analysis. A product-specific flavor wheel (5 modalities, 136 terms) with 17 key descriptors was built via Quantitative Descriptive Analysis. GC-O combined with AEDA/AECA identified 13 key aroma compounds in the commercial sample. HS-SPME-GC-MS detected 73 volatiles across all samples, among which olefins (34 species) were dominant and their formation was linked to lipid oxidation and high-temperature processing. Odor activity values and sensory data revealed that a frying temperature of 180 °C promoted nonanal and (E)-β-ocimene to enhance “fried seafood aroma”; Xiaomila chili pepper boosted “initial spiciness” via capsaicin; and high Sichuan pepper masked “fishy off-flavor” via linalyl acetate. A prediction model for aroma sensory attributes was established and the prediction correlations for “braised beef in soy sauce aroma” and “fried seafood aroma” were relatively high (r = 0.90 and 0.96, respectively). This study provides theoretical guidance for the flavor improvement of spicy anchovies.

## 1. Introduction

In the snack food market, flavored cooked fish and meat snacks are highly favored by consumers for their unique flavor and taste feel. Among these snacks, spicy anchovies—by virtue of the high-quality protein from deep-sea anchovies and their rich spicy flavor characteristics—have become one of the representative products in this category. This is a deeply processed food refined through multiple complex processes, including raw material selection, soaking and cleaning, frying, marinating, and seasoning mixing. Its flavor not only reflects the product’s core competitiveness but also acts as a key factor that influences consumers’ purchasing decisions and determines market acceptance [1]. Minor adjustments to different processing parameters, such as variations in frying temperature and changes in spice ratios, can significantly alter the product’s sensory attributes including aroma and taste through chemical processes like the Maillard reaction and lipid oxidation [2,3,4]. Therefore, in-depth analysis of the flavor formation mechanism of spicy anchovies and the rules regarding the impact of processing techniques bears important practical significance for promoting the standardized production of the product and improving quality stability. This also endows specialized research on spicy anchovies with clear market value and application demand.

In recent years, certain progress has been achieved in academic research on anchovy processing and flavor. However, obvious limitations still exist in the overall research. At the basic research level, existing achievements mostly focus on the analysis of approximate composition and amino acid composition of anchovies [5]. Early studies have explored the flavor profile changes in *Engraulis encrasicholus* during ripening [6]. They revealed that lipid oxidation-derived volatile compounds were key contributors to the characteristic umami and fermented aroma of cured anchovies, with aldehydes and ketones showing the most significant accumulation in the middle and late ripening stages. For drying processes, a study investigated intermittent microwave drying of anchovies and found that controlled microwave power (300–500 W) could modulate the degradation of flavor precursors (e.g., free amino acids and nucleotides), thereby enhancing the roasted and seafood-like aroma while inhibiting the formation of fishy off-flavor [7]. In terms of salted anchovy processing, Czerner et al. [8] demonstrated that ripening of *Engraulis anchoita* led to a decrease in polyunsaturated fatty acids (from 4.27 to 2.00 g kg^−1^) and an increase in thiobarbituric acid reactive substances (TBARS), but such lipid oxidation did not cause sensory deterioration and instead promoted the formation of the product’s typical pink color and cured flavor. In addition, explorations have been conducted from single dimensions, such as the nutritional characteristics of enzymatic hydrolysates [9] and quality changes during frozen storage [10]. Although these studies have provided partial theoretical support for anchovy processing, they do not extend the research perspective to the synergistic effects of multiple processes in actual production. For instance, fish embryo treatment, frying process, and seasoning formula, as key links in the production of spicy anchovies, have close flavor correlations with each other. Yet, there is currently a lack of systematic exploration into the laws of flavor evolution under the combined action of these links. Therefore, it is difficult to fully reveal the internal logic between process parameters and flavor formation, resulting in a disconnect between research findings and the actual needs of production. The production of spicy anchovies involves processes including fish embryo pretreatment, frying, stewing in soy sauce, and seasoning mixing. By adjusting each process parameter to prepare samples using the single-variable method, and analyzing their aroma and sensory differences, the impact of processes on flavor can be accurately revealed, which provides theoretical support for solving practical production problems.

In the field of sensory analysis, the flavor wheel is an important tool for visualizing sensory characteristics and building a communication bridge between producers and consumers. To date, it has been widely applied in flavor research of various foods such as meat [11], fruits [12], and seasonings [13]. However, for complex-flavored cooked fish snacks like spicy anchovies, the construction of their exclusive flavor wheel remains in a blank state. This leaves sensory evaluation without a unified and scientific analytical framework, making it impossible to accurately quantify the flavor differences of products. Quantitative Descriptive Analysis (QDA) is a method that systematically evaluates the sensory attributes of food through a well-trained panel [14]. It uses unified and precise descriptive terms, combined with quantitative scoring (e.g., a 9-point scale). By establishing a unified terminology database and reference substances, QDA not only covers multi-dimensional sensory attributes but also reduces evaluation subjectivity and features a high degree of standardization. In addition, its scoring system is highly quantitative, enabling it to accurately present sensory differences between different samples. All these provide data support for subsequent tasks, including the creation of a flavor wheel, the descriptive analysis of sample differences, the correlation of flavor substances, and the optimization of processes.

In the field of aroma compound identification, while significant advancements have been made in analytical technologies, critical gaps remain in their application to spicy anchovies. Gas Chromatography–Mass Spectrometry (GC-MS) and Gas Chromatography–Ion Mobility Spectrometry (GC-IMS) were the most widely used techniques for analyzing volatile compounds in aquatic products. For instance, Qiu et al. [15] employed GC-MS to investigate the effect of drying temperatures on volatile profiles in semi-dried golden pompano, identifying aldehydes and ketones as key contributors to lipid oxidation-derived aromas. Similarly, Zhang et al. [16] used GC-IMS to monitor volatile changes in dry-cured fish during storage, enabling rapid detection of off-flavor compounds like trimethylamine. However, these techniques primarily focused on the qualitative and quantitative analysis of volatile components and lacked the ability to directly link these compounds to sensory perception. This limitation restricted their utility in identifying aroma-active compounds which contribute to the actual perceived aroma. To address this limitation, Gas Chromatography–Olfactometry (GC-O) have been integrated with GC-MS to bridge chemical analysis and sensory experience. GC-O allowed trained panelists to detect and describe odor-active compounds as they elute from a gas chromatograph, enabling the identification of compounds that directly influence aroma [17]. Two common GC-O-based methods—Aroma Extract Dilution Analysis (AEDA) and Aroma Extract Concentration Analysis (AECA)—were applied to enhance the specificity of aroma-active compound identification. AEDA involved gradient dilution of aroma extracts until no odor was detected, with the Flavor Dilution (FD) factor indicating the relative importance of each compound [18]. It was particularly effective for identifying compounds with low olfactory thresholds (e.g., linalool, furfuryl alcohol), as these compounds remained detectable even after multiple dilutions [19]. However, AEDA failed to detect high-threshold compounds, as their concentrations became too low to perceive following dilution. In contrast, AECA operated in the reverse manner, it concentrated aroma extracts incrementally and recorded the minimum volume at which a compound was detected [20]. This method excelled at identifying high-threshold compounds (e.g., sabinene, γ-terpinene) but risked destroying thermally unstable or highly volatile low-threshold compounds during concentration [21]. Therefore, combining both methods with GC-O simultaneously enabled the realization of complementary advantages.

Furthermore, some studies had attempted to correlate volatile profiles with sensory attributes using multivariate statistical analysis. However, few have established robust predictive models for sensory aroma [22,23,24]; a limitation exacerbated by the reliance on traditional human sensory evaluation in flavor research. Traditional sensory evaluation of aroma required a trained panel to score product attributes based on subjective perception in accordance with standardized protocols [11]. Although traditional sensory evaluation could capture real sensory experiences, it has inherent drawbacks such as being time-consuming, labor-intensive, and susceptible to interference. Most importantly, it cannot establish a direct and quantifiable correlation between chemical compositions and sensory perception. Partial Least Squares Regression (PLSR) was a powerful tool for this purpose, as it could handle collinearity between volatile compounds and quantify their contributions to specific sensory attributes. For example, Yang et al. [25] used PLSR to link volatile compounds in black tea milk tea to sensory attributes like “roasted aroma” and “sweetness,” achieving correlation coefficients (r) > 0.8 for key attributes. In spicy anchovies, the aroma was complexly influenced by interactions between spice components and processing byproducts, among other factors. Therefore, PLSR could effectively integrate these complex interactions, realize the direct correlation between volatile substances and aroma sensory attributes, and further achieve the goal of predicting aroma sensory properties through volatile substances.

In this study, QDA was used to construct a flavor wheel for spicy anchovies. Additionally, the sensory differences between experimental samples and commercial products were compared, and a standardized sensory evaluation system was established. AEDA and AECA were combined with GC-O to identify the key aroma compounds of commercial products. Meanwhile, HS-SPME-GC-MS was employed to analyze the aroma compounds of all samples, thereby clarifying the effect of process variations on aroma composition. PLSR analysis was further conducted to establish a correlation model between volatile substances and sensory attributes, realizing the prediction of sensory characteristics. This research is expected to comprehensively establish a flavor database for spicy anchovies, providing theoretical support for the optimization of product production technology and the selection of raw materials and ingredients. At the same time, it will offer guidance for the development and production of other fish products, promoting technological progress and quality upgrading in the cooked fish and meat snack industry.

## 2. Results and Discussion

### 2.1. Defining the Sensory Phenotype of Spicy Anchovies Through Quantitative Descriptive Sensory Analysis

#### 2.1.1. Construction of the Flavor Wheel for Spicy Anchovies

**Figure 1:**
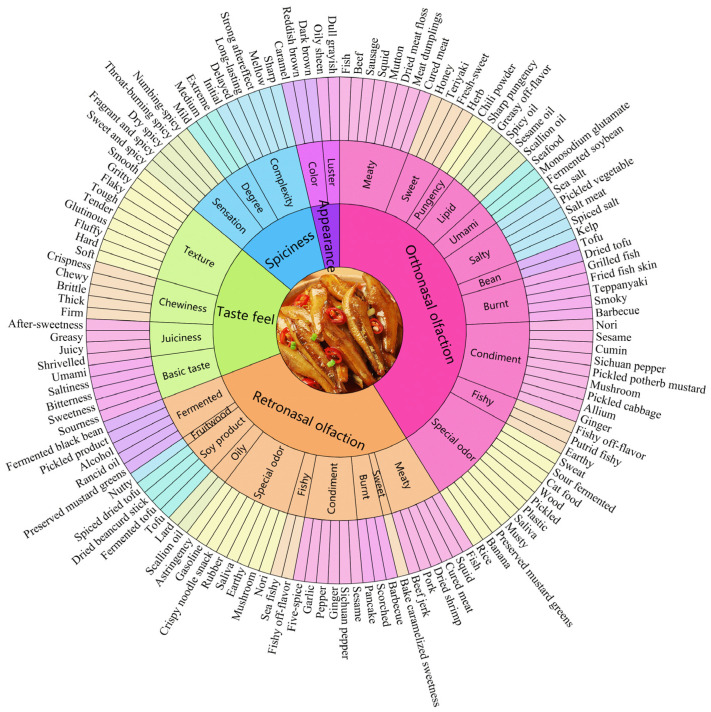
Flavor wheel of spicy anchovies. It includes all possible sensory attributes that spicy anchovies may produce. The flavor wheel consists of three layers: the innermost layer includes 5 main modalities, the middle layer contains 30 secondary terms, and the outermost layer comprises 136 specific descriptive terms.

To comprehensively understand the sensory characteristics of spicy anchovies, a flavor wheel for spicy anchovies was established. The flavor wheel for spicy anchovies comprised 136 sensory descriptive terms, including 56 aroma descriptors, 43 taste descriptors, 18 taste feel descriptors, 14 spiciness descriptors, and 5 appearance descriptors. The panel members discussed and established 5 main sensory modalities: orthonasal olfaction, retronasal olfaction, taste feel, spiciness, and appearance. We classified the descriptive terms according to these 5 modalities and formulated secondary terms. The secondary terms for orthonasal olfaction can be divided into 11 categories: meaty aroma, sweet aroma, pungency aroma, lipid aroma, umami aroma, salty aroma, bean aroma, burnt aroma, condiment aroma, fishy smell, and special odor. The secondary terms of retronasal olfaction include 10 categories: meaty taste, burnt taste, condiment taste, fishy taste, special odor, sweet taste, oily taste, soy product taste, fruitwood taste, and fermentation taste. The secondary terms of taste feel consist of 4 categories: texture, chewiness, juiciness, and basic taste. The secondary terms of spiciness include 3 categories: complexity, degree, and sensation. Appearance has 2 secondary terms: luster and color. At this point, the construction of the flavor wheel for spicy anchovies was completed (Figure 1).

In general, the flavor wheel for spicy anchovies consists of three levels, with 5, 30, and 136 descriptors from the innermost to the outermost layer, respectively, and the outermost layer represents clear and specific sensory characteristics. As can be seen from the results of the M-value calculation (Table S2), in terms of appearance, “oily sheen” and “caramel” were frequently mentioned, indicating that these are the main appearance characteristics of spicy anchovies. For aroma, “fish”, “sesame oil”, “grilled fish”, “fermented soybean” and “chili powder” had high mention frequencies, suggesting that these are important aroma characteristics of spicy anchovies. In terms of taste, “fish”, “saltiness”, “umami”, and “fishy off-flavor” were frequently mentioned, which are considered important taste characteristics of spicy anchovies. For texture and spiciness, “juicy”, “firm”, “chewy”, “fragrant and spicy”, and “medium spiciness” were frequently mentioned. This flavor wheel covers all possible sensory attributes of spicy anchovies. Even some difficult-to-perceive attributes such as “rancid oil”, “saliva”, and “earthy” were mentioned by evaluators. The development of the flavor wheel enabled the sensory characteristics of spicy anchovies to be presented more clearly and intuitively.

#### 2.1.2. Comparative Assessment of the Sensory Characteristic in Spicy Anchovies

QDA was used to analyze the flavor profile characteristics of 21 spicy anchovy samples, and 20 spicy anchovy samples were compared with commercial products to explore the sensory differences caused by processing changes. The M-value was calculated as the geometric mean of the frequency of each descriptor mentioned by the sensory panel and the intensity score of the descriptor. After calculating the M-value method (Table S2), descriptors with an M-value greater than the average of all M-values are retained. After principal component analysis (PCA) and hierarchical cluster analysis (HCA), descriptors with similar meanings were further deleted and merged. Finally, after discussion among the panelists, 17 sensory descriptive terms were selected for sensory evaluation of the spicy anchovy samples. They were oily sheen, brown, fried seafood aroma, fish meat aroma, braised beef in soy sauce aroma, cured meat aroma, sweetness, umami, fishy off-flavor, saltiness, braised sauce taste, chewiness, juiciness, toughness, initial spiciness, delayed spiciness, and strong aftertaste.

**Figure 2:**
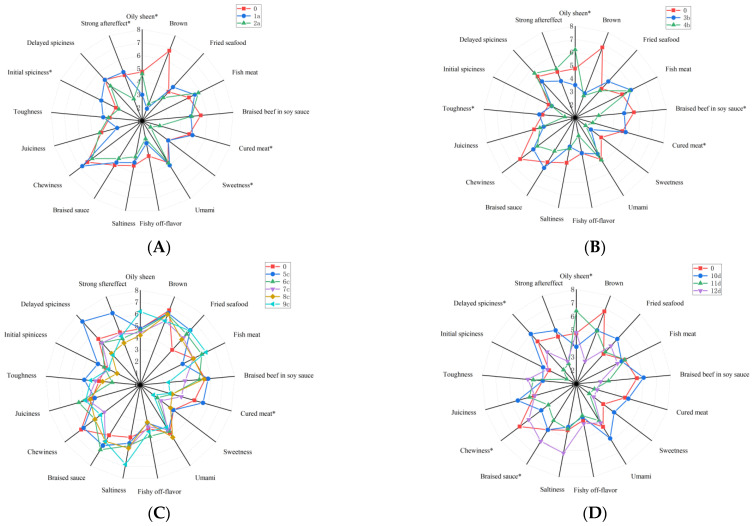

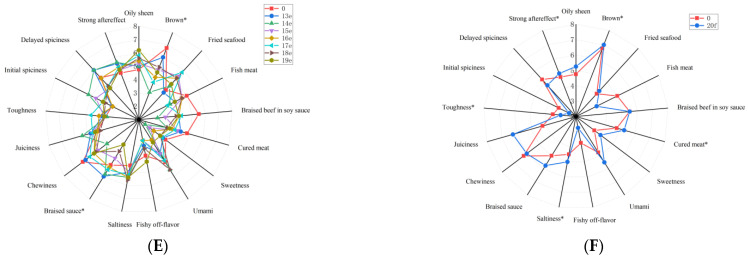
Sensory evaluation results of different samples of spicy anchovies. Comparison of the samples with modified same-type processes together with the commercial spicy anchovy products. * indicates significance at *p* ≤ 0.05. 0 refers to commercial spicy anchovy products; (**A**) 1a and 2a are samples with modified additive-treated fish embryos; (**B**) 3b and 4b are samples with modified frying process; (**C**) 5c, 6c, 7c, 8c and 9c are samples with modified chili oil recipe; (**D**) 10d, 11d and 12d are samples with modified preparation method of chili oil; (**E**) 13e, 14e, 15e, 16e, 17e, 18e and 19e are samples with modified seasoning paste; (**F**) 20f is a sample with modified seasoning powder.

The sensory evaluation results of different samples of spicy anchovies were shown in Figure 2. The additives used in this study were mainly protease and lipase. Samples with modified additive-treated fish embryos exhibited significant differences in the sensory characteristics of oily sheen, cured meat aroma, sweetness, initial spiciness, and strong aftertaste compared with commercial products (*p* ≤ 0.05). Sample 1a had prominent sensory characteristics such as fried seafood aroma, cured meat aroma, umami, chewiness, toughness, initial spiciness, and strong aftertaste. Sample 2a had prominent sensory characteristics of fish meat aroma and juiciness. It could be observed that the method of treating fish embryos with lipase, which was adopted for Sample 1, could enhance more sensory characteristics. The degradation of fats into fatty acids and glycerol was catalyzed by lipase. On one hand, more lipid oxidation precursors such as oleic acid were released, which enhanced the fried seafood aroma and oil brightness. On the other hand, excessive degradation would lead to fat loss, by which juiciness was reduced and the toughness texture was increased. Sample 2a was prominent in fish meat aroma and juiciness, which was attributed to the fact that fish meat proteins were hydrolyzed by proteases to generate small-molecule peptides and free amino acids (e.g., glutamic acid). The meat texture was loosened and water retention was improved through such moderate hydrolysis. Meanwhile, the Maillard reaction was promoted by the increase in amino acids, by which the meat aroma and umami taste were strengthened. In addition, some commonly used additives such as sodium acetate and sodium lactate can also affect the shelf life, chemical quality, and sensory attributes of products during storage [26].

The change in frying process in this study was specifically reflected in temperature. Samples with modified frying process exhibited significant differences in the sensory characteristics of oily sheen, braised beef in soy sauce aroma, cured meat aroma, and toughness compared with commercial products (*p* ≤ 0.05). Sample 3b had prominent sensory characteristics such as fried seafood aroma, fish meat aroma, cured meat aroma, braised sauce taste, and toughness. Sample 4b had prominent sensory characteristics of oily sheen, delayed spiciness, and strong aftertaste. It was observed that the frying temperature of 180 °C used for Sample 3b was more effective in enriching the product’s aroma. Previous studies have indicated that under different frying temperatures (160–220 °C), reducing sugars and amino acids undergo the Maillard reaction, generating a variety of volatile compounds such as pyrazines and furans [27]. These volatile compounds endow the product with aroma characteristics like burnt, nutty, and toast-like notes. The frying temperature for Sample 3b was approximately 180 °C. At this temperature, aldehydes and olefins (e.g., nonanal, (E)-β-ocimene) produced by fat oxidation were dominant. The Maillard reaction progresses at different rates across 120–220 °C. It proceeds slowly below 140 °C and markedly accelerates between 140 and 180 °C. Above 200 °C, violent pyrolysis tends to generate excessive burnt by-products. Since the maximum temperature reached 220 °C, the conditions at 180 °C can be considered relatively mild, thereby avoiding the development of a strong burnt aroma. Such a temperature made the fried seafood aroma and fish meat aroma of Sample 3b prominent. It not only retained the inherent base aroma of seafood but also enhanced the characteristic fried aroma through moderate lipid oxidation.

The changes in the chili oil recipe in this study were mainly the use of different types of chili peppers and Sichuan peppercorns. Samples with modified chili oil recipe exhibited significant differences in the sensory characteristics of cured meat aroma compared with commercial products (*p* ≤ 0.05). Sample 5c had prominent sensory characteristics such as braised beef in soy sauce aroma, cured meat aroma, toughness, initial spiciness, delayed spiciness, and strong aftertaste. Sample 6c had prominent sensory characteristics of fishy off-flavor, braised sauce taste, and juiciness. Sample 7c had relatively balanced sensory characteristics. Sample 8c had a prominent umami sensory characteristic. Sample 9c had prominent sensory characteristics of oily sheen, fish meat aroma, and saltiness. It can be seen that the chili oil recipe of Sample 5c not only enriched and enhanced the aroma but also strengthened the overall spiciness of the product. Sample 5c achieved excellent performance because Xiaomila (a type of spicy chili pepper) was selected. This type of chili pepper has a pleasant aroma and a relatively high spiciness level [28]. In addition, sample 9c was prominent in fish meat aroma and low in fishy odor, which was consistent with the result that a relatively high amount of Sichuan pepper was used. This was because citronellol in Sichuan pepper was able to mask the fishy odor and endow the product with a numbing taste [29].

This study used oil pouring or frying methods to prepare chili oil. Samples with modified preparation method of chili oil exhibited significant differences in the sensory characteristics of oily sheen, braised sauce taste, chewiness, and delayed spiciness compared with commercial products (*p* ≤ 0.05). Sample 10d had prominent sensory characteristics such as fried seafood aroma, braised beef in soy sauce aroma, cured meat aroma, sweetness, umami, juiciness, initial spiciness, delayed spiciness, and strong aftertaste. Sample 11d had a prominent oily sheen sensory characteristic. Sample 12d had prominent sensory characteristics of fishy off-flavor, saltiness, braised sauce taste, and toughness. Results showed that the chili oil brewing method of Sample 10d was relatively optimal. The chili oil of Sample 10d was prepared by frying, a method similar to high-temperature immersion. During this process, spices fully released fat-soluble components in the high-temperature oil, such as eucalyptol from Sichuan pepper and capsaicin from chili peppers. In addition, the frying method enabled the umami peptides from the beef paste in the chili sauce to blend with the pungent aroma of spices, forming a mellow and complex flavor. This ultimately resulted in Sample 10d being prominent in lingering spiciness, strong aftertaste, and sauced beef aroma. The chili oil of Sample 11d was prepared by pouring, a method more akin to high-temperature instantaneous pouring. Although this method released more volatile aroma substances, it led to less dissolution of capsaicin. Therefore, Sample 11d exhibited a prominent oily sheen and mild spiciness. This was because the oil-pouring method reduced oil oxidation, enabling more undegraded oil to be retained on the surface. Meanwhile, the release of spicy substances was insufficient.

Seasoning paste mainly consists of protein extracts, seasonings (salt, sugar, spices, etc.), additives (umami agents, antioxidants), and thickeners. Differences in the amount of protein extracts or in fermentation processes contribute to the flavor variations of seasoning pastes [9,30]. Beef paste and chicken paste were mainly used in this study, and their impact on sensory properties was explored. Samples with modified seasoning paste exhibited significant differences in the sensory characteristics of brown color and braised sauce taste compared with commercial products (*p* ≤ 0.05). Sample 13e had a prominent braised sauce taste sensory characteristic. Sample 14e had prominent juiciness and initial spiciness sensory characteristics. Sample 17e had prominent fried seafood aroma and toughness sensory characteristics. Sample 19e had prominent oily sheen and fishy off-flavor sensory characteristics. Samples 15e, 16e, and 18e had relatively balanced sensory characteristics. It can be seen that except for Sample 19e, which increased the fishy off-flavor and had a negative impact on the product, the other samples had their own characteristics. Further selection can be carried out according to consumer preference surveys in the future. Inosinic acid and peptides in beef can enhance braised beef in soy sauce aroma and braised sauce taste. Moreover, succinic acid and glutamic acid in beef synergize with the soy sauce flavor in the halogenation process to strengthen the salty and rich taste. Sample 13e had the most prominent braised sauce taste, which was due to the use of a high proportion of beef paste. The sensory characteristics of Samples 15e and 16e were well-balanced, which was precisely because the mixture of chicken paste and beef paste prevented the excessive intensity of a single meat aroma. In addition, the degradation products of chicken protein (e.g., IMP) endowed the product with a milder meat aroma and umami taste.

Samples with modified seasoning powder exhibited significant differences in the sensory characteristics of brown color, cured meat aroma, saltiness, toughness, and strong aftertaste compared with commercial products (*p* ≤ 0.05). Sample 20f had prominent sensory characteristics such as brown color, fried seafood aroma, cured meat aroma, sweetness, umami, saltiness, braised sauce taste, juiciness, strong aftertaste, and oily sheen. It can be seen that this seasoning powder was better than commercial products because it made the taste richer and more intense. Bacon aroma was provided by beef powder, and the Maillard reaction was promoted by sucrose, which generated 3-furanmethanol (with fruity and sweet aromas) [31,32]. Specifically, under thermal processing conditions, sucrose, as a non-reducing disaccharide, was first hydrolyzed to produce glucose and fructose. The fructose was then preferentially subjected to 1,2-enolization, forming 3-deoxyglucosone—a critical intermediate that was further degraded into 3-furanmethanol. The generated glucose was involved in the reaction with amino groups from beef powder-derived amino acids or proteins, which initiated and facilitated the Maillard reaction. The whole process ultimately contributed to the formation of the target fruity-sweet aroma compound. The appropriate ratio of the beef powder and sucrose enhanced the characteristic flavor of cured meat products.

In conclusion, by using proteases and lipases, controlling the frying temperature precisely, adjusting the formula or preparation method of chili oil, and selecting raw materials for seasoning pastes and powders, the protein degradation, fat oxidation, the Maillard reaction and the release of spice components could be regulated. It was precisely these regulations that ultimately led to obvious differences in appearance, aroma, taste, texture and spiciness among different spicy anchovy samples. The directional adjustment of these process parameters provided operable regulatory targets for the flavor standardization of spicy anchovies.

### 2.2. Characterization of Key Aroma Components in Spicy Anchovies

To identify the key characteristic volatile substances in spicy anchovies, a representative commercial spicy anchovy product was selected for detection and analysis in this experiment. GC-O analysis was conducted by combining the AEDA method and AECA method, with a focus on the aroma of the products. Preliminary test analysis showed that the types of volatile flavor substances extracted by the two pretreatment methods, SPME and SAFE, were almost the same. Therefore, SAFE was chosen to collect volatile flavor substances, which facilitated the concentration or dilution of the solution. The results are shown in Table 1. A total of 28 substances were detected, among which 13 substances with FD ≥ 4 and volume ≥ 10 mL were identified as key aroma compounds through the joint identification of AEDA and AECA. These substances were D-limonene, cis-anethole, terpinolene, diacetone alcohol, 4-terpineol, 3-furylmethanol, linalyl acetate, octaethylene glycol monododecyl ether, 2-methoxy-5-(prop-2-enyl)phenol, ethyl maltol, anethole, 2-acetylpyrazine, and 4-allylanisole.

D-limonene mainly stemmed from citrus spices (such as lemon peel and orange peel). Cis-anethole, anethole, and 4-allylanisole mainly existed in star anise and fennel. These substances can be directly released from spices via frying or baking during the processing of spicy anchovies, primarily contributing lemon, fennel, pungent, and herbal aromas. Terpinolene and 4-terpineol are common in herbs such as mint and pine needles, and also in fruits [33], mainly providing grassy and malt aroma. During the frying process, terpinolene may undergo oxidative degradation to generate aldehydes or ketones. 3-Furylmethanol and 2-acetylpyrazine were both associated with the Maillard reaction. They were generated via the reaction between amino acids and reducing sugars, and served as sources of burnt, roasted, and sweet aromas [34]. Linalyl acetate has floral and citrus aromas. It was a key aroma compound in Sichuan pepper [29], which masked the inherent fishy smell of anchovies and enhanced the overall aroma harmony. Ethyl maltophenol enhanced caramel aroma and sweetness, and at the same time inhibited bitter substances (such as alkylpyridine compounds) produced by the Maillard reaction [35]. 2-Methoxy-5-(prop-2-enyl)phenol may be a derivative of eugenol, existing in spices such as clove and cinnamon. In addition, diacetone alcohol was probably solvent residue, and octaethylene glycol monododecyl ether stemmed from additives (emulsifiers or stabilizers) in the product.

It was worth mentioning that 10 substances, including sabinene, γ-terpinene, α-curcumene, zingiberene, 2-carene, (-)-4-terpineol, triethyl phosphate, 2-methyl-5-isopropyl-bicyclo[3.1.0]hexan-2-ol, cis-2-(2-pentenyl)furan, and 2-acetylpyrrole, can only be detected by the AECA method. This was possibly because they had extremely high olfactory thresholds. Thus, even if their concentration was relatively high during the dilution process of AEDA, they cannot be detected by the human sense of smell. However, the AECA method further concentrates the sample via a concentration step. Thus, these substances can be detected once their concentration reaches the instrument’s detection limit. Three substances, linalool, furfuryl alcohol, and 4-methyl-5-hydroxyethylthiazole, can only be detected by the AEDA method. This is possibly because they have extremely low olfactory thresholds. Thus, they can be detected by the human sense of smell even after continuous dilution. Moreover, due to their high volatility (for example, linalool has a boiling point of about 198 °C) or thermal instability (for example, furfuryl alcohol is easily oxidized), they are destroyed or escaped during the AECA concentration process, resulting in failure to detect them when using the AECA method. The phenomenon that these substances can only be detected by one method indicates that the two methods complement and improve each other to a certain extent; that is, using the two methods in combination will yield more comprehensive results.

### 2.3. The Impact of Process Changes on the Volatile Compounds of Spicy Anchovies

Through GC-O analysis combined with the AEDA and AECA methods, Section 2.2 has identified 13 key aroma compounds from commercial spicy anchovy products. These compounds derived from spice release, lipid oxidation, and Maillard reactions. They were the core substances that form the characteristic flavors of the product, such as pungent, anise, and burnt aromas. However, the flavor characteristics of commercial products were determined by specific process conditions. Given that key process parameters (including fish pretreatment, frying process, and chili oil formulation) may be modified, it is necessary to clarify whether such modifications will alter the content of these key aroma compounds and the composition of other volatile components. It was found in the preliminary experiment that the volatile substances detected after the two pretreatment methods—namely, SPME and SAFE—were basically the same. Since the number of samples to be tested was relatively large, SPME was selected as the collection method for volatile substances to ensure convenient and efficient detection. To systematically reveal the intrinsic relationship between processes and flavor, SPME combined with GC-MS analysis was further used to identify the aroma compounds in 21 spicy anchovy samples (including commercial products). The content variation patterns of compounds such as olefins, alcohols, and phenols under different process conditions were investigated to clarify the specific impact mechanism of process changes on the aroma composition of spicy anchovies.

**Figure 3:**
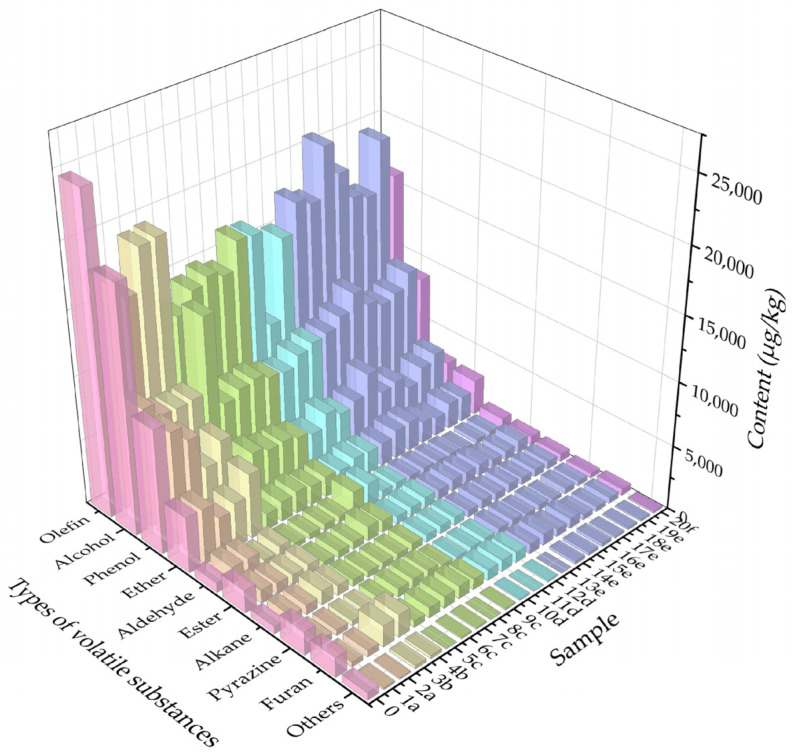
Total content of various aroma compounds in spicy anchovies.

**Figure 4:**
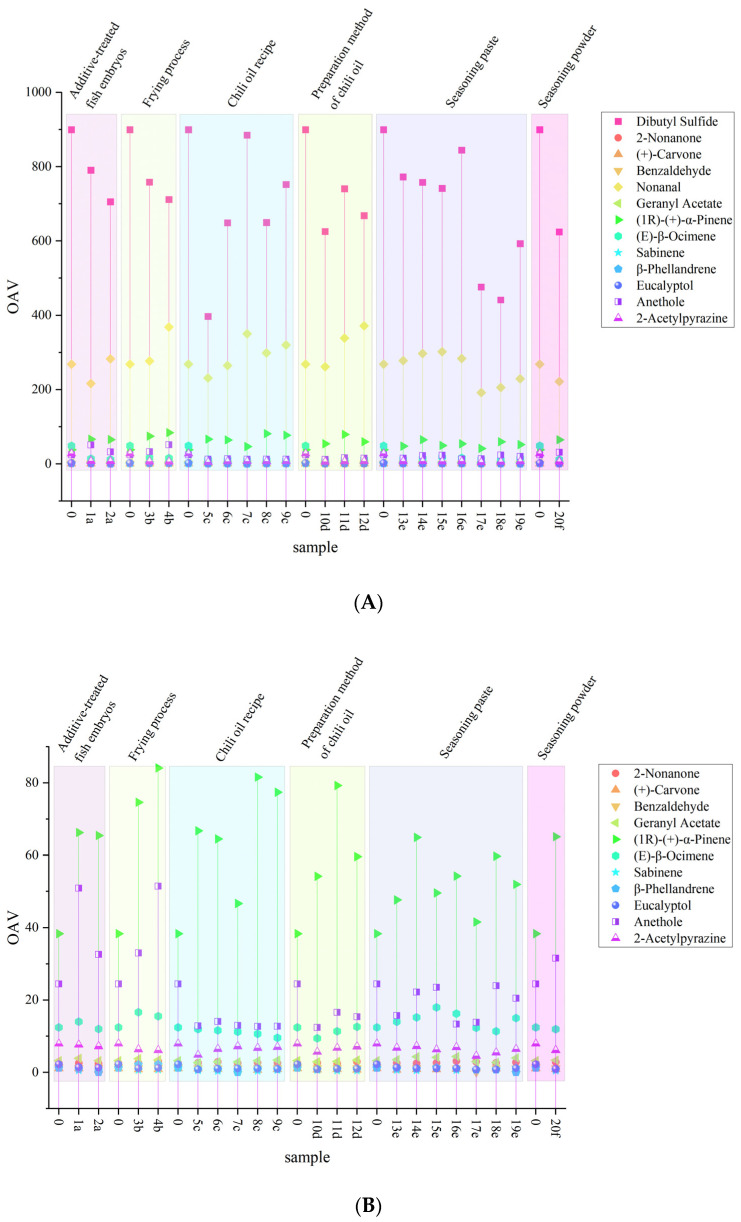
(**A**) Comparison Chart of OAVs for Various Substances. Individual substances detected only in two or three samples are regarded as impurities and not included in the discussion. Sample 0 refers to the commercial spicy anchovy products. (**B**) Results when removing the two substances with relatively high values: dibutyl sulfide and nonanal.

Volatile substances with a qualitative value ≥ 60 were selected for quantitative analysis. A total of 73 odor compounds were identified in all samples, and these compounds were further classified into 10 chemical categories: olefins, alcohols, phenols, ethers, aldehydes, esters, alkanes, pyrazines, furans, and others. Among them, olefins accounted for the largest number (34 species), followed by alcohols (10 species), esters and aldehydes (5 species each), phenols (4 species), and ketones (3 species), with ethers being the least (2 species). The total content of each category of compounds across all samples was visualized in Figure 3. The OAV can quantify the contribution of odor compounds to the overall aroma of spicy anchovies. The calculation results are listed in Table S3. For some substances (e.g., 2-methoxy-5-(prop-2-en-1-yl)phenol, 4-allylanisole), no olfactory threshold values were identified in the existing literature or experimental data. Thus, they are not included in the table. Substances such as 4-Isopropylbenzaldehyde, safranal, and carene were only detected in very few samples, indicating that their formation has no stable correlation with core process parameters like fish embryo treatment, frying process, and chili oil formulation. Instead, they are more likely caused by accidental factors such as single raw material contamination, reagent residue, or instrument error, and thus cannot reflect the intrinsic relationship between processes and flavor. Therefore, these substances are regarded as impurities and excluded from the discussion. It has been pointed out that compounds with OAV > 1 are considered to contribute to the aroma of samples. Statistics showed that 13 volatile substances with OAV > 1 were detected in almost all samples, namely dibutyl sulfide (OAV = 396.74–1386.04), 2-nonanone (OAV = 2.45–3.13), (+)-carvone (OAV = 0.64–1.07), benzaldehyde (OAV = 1.16–1.48), nonanal (OAV = 192.08–371.5), geranyl acetate (OAV = 2.68–4.41), (1R)-(+)-α-pinene (OAV = 38.3–84.12), (E)-β-ocimene (OAV = 9.42–17.96), sabinene (OAV = 0.35–1.42), β-phellandrene (OAV = 0.7–2.21), eucalyptol (OAV = 0.79–2.24), anethole (OAV = 12.41–51.43), and 2-acetylpyrazine (OAV = 4.55–7.93). These substances mainly provide pungent, herbal, sweet, fruity, roasted, and slight floral aromas. The OAVs of these substances in each sample were compared with those of commercial products and presented in Figure 4. Due to the substantial differences in OAVs between dibutyl sulfide, nonanal and other substances, plotting them on the same coordinate system would make it impossible to distinguish the OAVs of other substances. Therefore, dibutyl sulfide and nonanal were excluded in plotting Figure 4B to facilitate better comparison of the other substances. As shown in Figure 4, the OAVs of five substances (dibutyl sulfide, nonanal, (1R)-(+)-α-pinene, anethole, and (E)-β-ocimene) were significantly higher than those of other substances. This is mainly because these substances have low threshold values, which leads to high OAVs.

Regarding the olefins in this study, olefins were found to have the highest content among all other samples except Sample 5c (Figure 3). Volatile olefins usually have fatty and herbal aromas. An appropriate amount can enhance the seafood flavor and burnt aroma of the product. However, an excessive amount may lead to flavor deterioration like rancidity. There may be three reasons for the predominance of olefins. First, anchovies themselves are rich in fatty acids and phospholipids [9], which easily generate olefins after oxidation. Second, high-temperature drying and frying can accelerate lipid oxidation and Maillard reactions, promoting the release of olefins. Third, the superposition of vegetable oil oxidation and fish lipid reactions increases the total amount of olefins. As observed in Figure 4B, only (1R)-(+)-α-pinene and (E)-β-ocimene had an OAV > 1 in all samples, while sabinene and β-phellandrene had an OAV > 1 in only 7 samples. Specifically, the OAVs of (1R)-(+)-α-pinene in Samples 4b, 8c, and 11d were the highest among all samples (Figure 4B). It can be concluded that frying at 200 °C, addition of a high proportion of Sichuan pepper, and oil-pouring method for chili oil preparation increased the OAV of (1R)-(+)-α-pinene. Meanwhile, the OAV of (1R)-(+)-α-pinene in all samples was higher than that in commercial products. Thus, all process changes increased the content of (1R)-(+)-α-pinene and also imparted the rancid and smoky flavors, which also explains why these terms appeared in the sensory evaluation. This terpene compound underwent thermal degradation under frying conditions (180–200 °C), and its degradation products include p-cymene, limonene, and α-terpineol. These degradation products were also detected in the samples and may further impart smoky and roasted flavors to fried foods through Maillard reactions or lipid oxidation [36]. As observed in Figure 4B, the OAVs of (E)-β-ocimene in Sample 15e were the highest among all samples. It can be concluded that the ratio of seasoning pastes played a significant role in the formation of (E)-β-ocimene. It was inferred that the high proportion of chicken paste promoted the formation of (E)-β-ocimene, leading to a more harmonious balance between fishy aroma and herbal aroma in Sample 15e. In addition, Samples 3b and 4b with modified frying processes showed a higher OAV of (E)-β-ocimene than commercial products. This may be attributed to the fact that frying process promoted the formation of (E)-β-ocimene through high-temperature-driven Maillard reaction, fatty acid oxidation, and precursor release. (E)-β-ocimene exhibits herbal and floral aromas and is present in over 70% of seed plant families [37]. It mainly comes from basil and perilla, and its significant contribution to the product flavor is likely due to the use of these two spices in the processing of spicy anchovies.

Alcohols are mainly derived from the oxidative decomposition of fats or the reduction of carbonyl compounds [17,38]. Generally, the threshold of saturated alcohols is higher than that of unsaturated alcohols. Therefore, unsaturated alcohols contribute more significantly to the flavor of samples. The main alcohols detected in this study were geraniol, linalool, and 4-terpineol. Among them, 4-terpineol was only detected in commercial products, Sample 17e, and Sample 18e. It was also one of the key aroma compounds jointly identified in commercial products via GC-O combined with AEDA and AECA methods. 4-Terpineol has a complex aroma of pine, green grass, and flowers. The reason why it was detected in Sample 17e and Sample 18e, where the seasoning paste was modified, may be that these two samples were added with spices containing terpene components such as mint and pine needles. Alternatively, it may be because the terpene precursors in the seasoning paste were accidentally subjected to enzymatic hydrolysis or moderate heating, which caused the precursors to degrade and introduce hydroxyl groups, ultimately generating 4-terpineol. However, linalool and geraniol have relatively high olfactory thresholds and low contents, resulting in OAV of less than 1. It was observed from Figure 3 that Sample 5c had the highest content of alcohols. It was hypothesized that Sample 5c (spiced with Xiaomila chili peppers) might contain more abundant alcohol precursors (such as fatty alcohols and terpene alcohol precursors). When mixed with other raw materials and heated, these precursors were more likely to be converted into free alcohols through reactions like hydrolysis and oxidation.

Esters are formed by the condensation of alcohols and fatty acids. Among them, lactones and thioesters contribute more to the meaty flavor than other types of esters [39]. In addition, esters have a characteristic woody and herbal aroma and are commonly found in lemongrass and cardamom, allowing them to play a role in seasoning [40]. In this study, the reason why geranyl acetate had an OAV > 1 was the use of lemongrass and cardamom. Among all samples, the OAVs of geranyl acetate in Samples 14e, 15e, and 16e (changed in seasoning pastes) were the highest. However, there was no significant difference across all samples. It is speculated that these process adjustments do not involve the three core links of “precursor supply”, “thermochemical reaction”, and “degradation inhibition” for ester substances. In subsequent studies, experiments can be designed based on these directions to verify their effects on esters.

Aldehydes are derived from the degradation of unsaturated fatty acids [41,42]. They are believed not only to help enhance the flavor of fish-based foods but also to generate flavor through the interaction of amino carbonyls with other compounds [43]. Studies have found that some aldehydes such as nonanal, hexanal, and benzaldehyde contribute significantly to the composition of meaty flavors [44]. In this study, the aldehydes with OAV > 1 were nonanal and benzaldehyde. Nonanal has a citrus-like and grassy aroma, which is mainly produced by the oxidation of oleic acid [45] and has been confirmed as a key aroma compound in fish meat [46]. Benzaldehyde has an almond and caramel aroma. As a degradation product of phenylalanine, it further indicated that the degradation of amino acids occurred during the Maillard reaction [47]. As observed in Figure 4, the OAVs of nonanal in Sample 4b (fried at 200 °C), Sample 7c (with Erjingtiao chili peppers), and Sample 12d (oil-soaking method) were the most prominent among all samples. This indicated that high temperatures accelerated the oxidation of oleic acid to form aldehydes. However, benzaldehyde had no significant difference across samples due to its low OAVs. It is speculated that the influencing factors may be the freshness and storage conditions of anchovy raw materials, which can affect the initial contents of fatty acids and amino acids. Anchovies with low freshness or excessively long frozen storage time undergo premature lipid oxidation, which may lead to an imbalance of aldehyde precursors in the raw materials, thereby affecting the contents of nonanal and benzaldehyde in the processed products.

Phenolic compounds are mainly generated through the thermal degradation of phenolic acids and lignin. They can also be formed by the action of microbial enzymes and ferulic acid, which is a precursor of phenolic compounds. Their flavor characteristics are smoky and pungent aromas [21]. Eugenol, carvacrol and ethyl maltol are the main phenolic substances detected in the samples. However, their OAVs were less than 1. Thus, they contribute insignificantly to the flavor of spicy anchovies.

Ketones are mainly produced through the thermal oxidation or degradation of unsaturated fatty acids, as well as the degradation of amino acids. Most of them have a rich and pleasant aroma [48]. Some ketones are important intermediates for the formation of heterocyclic compounds and play a key role in the development of meaty flavors [39]. The ketones detected in all samples with OAV > 1 were 2-nonanone and (+)-carvone. As shown in Figure 4B, there was no significant difference in their content across all samples. They are both important products of the Maillard reaction, fat oxidation, and lipid degradation. These reactions easily occur in products under high-temperature frying conditions. In addition to the processes mentioned in this study, the temperature and time during the sterilization process may alter the oxidation rate of residual unsaturated fatty acids and the amino acid degradation process in fish meat, thereby affecting the balance between the formation and degradation of ketone substances.

Furan compounds are mainly derived from the intermediates or final products of the Maillard reaction, and they are usually generated during sugar browning reactions and sugar degradation processes. In this study, 2-ethylfuran and 2-pentylfuran were detected. However, due to their low content and high threshold values, they contribute little to the product’s flavor.

Pyrazines, as typical Maillard reaction products, are major components in baked, roasted, and popcorn-based foods [49]. Alkylpyrazines are common volatiles in many heated foods, and roasted aromas typically come from alkylpyrazines [44]. In this study, tetramethylpyrazine, 2,3,5-trimethylpyrazine, and 2-acetylpyrazine were mainly detected. However, only 2-acetylpyrazine had an OAV > 1, and it mainly produces barbecue and baking aromas. Notably, as can be seen in Figure 4B, all process changes reduced the content of 2-acetylpyrazine to varying degrees. The OAVs of 2-acetylpyrazine in Samples 5c (Xiaomila chili peppers) and 17e (beef:chicken = 1:2) were the lowest among all samples. The reason might be that the relatively high capsaicin content in Xiaomila chili peppers indirectly inhibited the Maillard reaction. Additionally, the degradation products of chicken protein from the high-proportion chicken paste had a weaker promoting effect on the Maillard reaction than beef paste, resulting in a reduction in the production of 2-acetylpyrazine.

Ether compounds are mainly produced through the dehydration condensation of alcohol compounds. In the formation of food flavor, ethers not only act as volatile aroma components to directly contribute to flavor, but also serve as precursors in participating in the Maillard reaction or lipid oxidation process. They can mask unpleasant odors and impart a cool, aniseed, sweet, and herbal aroma to meat products. Eucalyptol, anethole, and dibutyl sulfide were three ether compounds that make significant contributions to the product (with OAV > 1). Eucalyptol and anethole have a pungent and herbal odor, mainly derived from star anise and fennel [50]. Plants containing anethole can also be used as spices, oral fresheners, and sweeteners [51]. The spices used in the production of spicy anchovies included fennel and star anise, which were the main reasons for the generation of eucalyptol and anethole. Observations revealed that the OAV of anethole in samples with modified processes (including anchovy embryo treatment with additives, adjustment of frying temperature, and modification of seasoning powder) was higher than that in commercial products. This indicated that modifications to these processes increased the content of anethole, likely because these processes were more effective at releasing the aroma compounds from the spices. At low concentrations, dibutyl sulfide exhibits a pungent odor similar to garlic and onion; at high concentrations, it produces an unpleasant rancid sulfur smell. Studies have pointed out that in fried foods, volatile sulfur compounds are positively correlated with oil temperature and frying time. In detail, high temperatures and long frying durations can lead to an increase in the type and concentration of sulfur compounds [52]. As can be seen in Figure 4B, the OAV of dibutyl sulfide in all samples was lower than that in commercial products, meaning all process changes have reduced the content of dibutyl sulfide. It was mainly because that all process changes indirectly inhibit its formation. First, some process adjustments involve modifying frying parameters, such as reducing frying temperature and shortening frying time, which reduces heat-driven reactions. This was consistent with the result that the OAV of dibutyl sulfide in Sample 3b (fried at 180 °C, OAV = 711.46) was lower than that in Sample 4b (fried at 200 °C, OAV = 758.25). Second, the enzymatic hydrolysis of fish embryos focuses on protein hydrolysis, which decreases the release of sulfur-containing precursors necessary for dibutyl sulfide synthesis. This was consistent with the result that the OAV of dibutyl sulfide in Sample 1a (treated with lipase, OAV = 790.42) was higher than that in Sample 2a (treated with protease, OAV = 705.21). Third, reducing the proportion of sulfur-containing seasonings and flavoring spices may not only inhibit the formation of dibutyl sulfide but also accelerate its degradation. The OAV of dibutyl sulfide in Sample 5c (spiced with Xiaomila chili peppers) was 396.74, the lowest among Samples 5c to 9c. This was precisely because the low oil content in Xiaomila chili peppers reduced the release of sulfur-containing precursors. Meanwhile, the lack of pungent aroma (garlic and onion aromas) also resulted in a slightly higher fishy odor in this sample.

### 2.4. Construction of Aroma Sensory Prediction Model Based on Volatile Flavor Compounds

#### 2.4.1. Analysis of Correlation Between Key Volatile Substances and Aroma Sensory Properties

**Figure 5:**
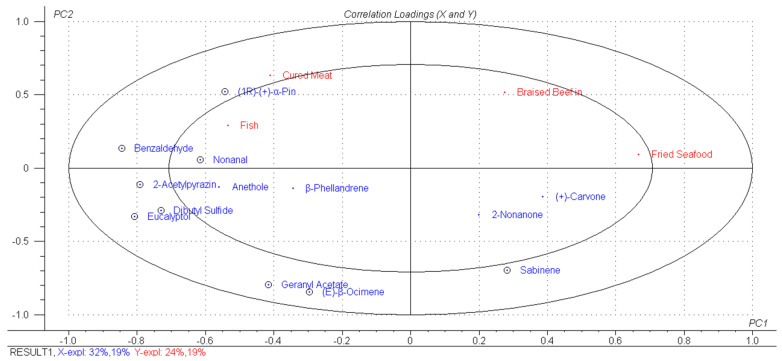
PLS2 correlation load diagram of PC1 and PC2 (the explained variance for X is 51%, and the explained variance for Y is 43%). The X variables (blue) are volatile substances with OAV > 1 and detected in most samples, and the Y variables (red) are aroma sensory attributes (fried seafood aroma, fish meat aroma, braised beef in soy sauce aroma, cured meat aroma). The ellipses represent R^2^ = 0.5 and 1.0, respectively.

The correlation between volatile substances and sensory properties was studied using PLSR. Taking volatile substances with OAV > 1 as X variables and aroma sensory attributes (fried seafood aroma, fish meat aroma, braised beef in soy sauce aroma, cured meat aroma) as Y variables, PLS2 analysis was performed. Before modeling, the volatile compound data (X variables) were subjected to autoscaling preprocessing, and the sensory data (Y variables) were subjected to mean centering preprocessing to eliminate the influence of different dimensions and magnitudes of data. The PLSR model included three significant principal components (PCs). Since no additional information was obtained through the examination of PC 2 and PC 3, only PC 1 and PC 2 were discussed. Figure 5 showed the correlation loading plot of PC1 and PC2. The ones marked with small circles in the figure represent significant variables, and the two large ellipses in the figure represent 50% and 100% of the explained variance of the PLSR model, respectively. The model explained 51% of the variance in X and 43% of the variance in Y. The main reason for the low explained variance was that the flavor of spicy anchovies was influenced by both volatile compounds and non-volatile taste-active substances (such as amino acids and nucleotides). However, this model only took volatile compounds into account, resulting in limited explained variance. Although the explained variance was not very high, 9 X variables that had significant correlations with Y variables were obtained, and most of them were located between the two ellipses; namely, (1R)-(+)-α-pinene, benzaldehyde, nonanal, 2-acetylpyrazine, dibutyl sulfide, eucalyptol, geranyl acetate, (E)-β-ocimene, and sabinene. In addition, three substances—(+)-carvone, 2-nonanone, and sabinene—were positively correlated with braised beef in soy sauce aroma and fried seafood aroma; while 10 substances—dibutyl sulfide, benzaldehyde, nonanal, geranyl acetate, (1R)-(+)-α-pinene, (E)-β-ocimene, β-phellandrene, eucalyptol, anethole, and 2-acetylpyrazine—were positively correlated with cured meat aroma and fish meat aroma.

**Figure 6:**
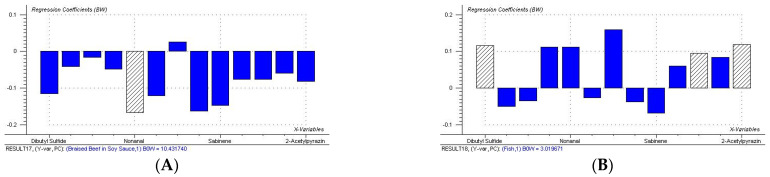

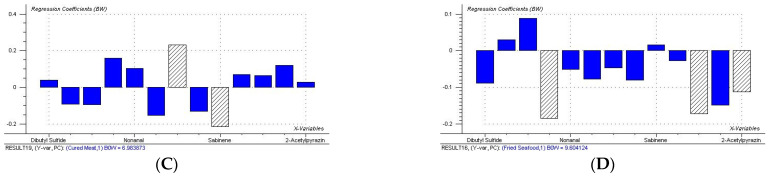
PLS1 contribution analysis of volatile compounds to braised beef in soy sauce aroma (**A**), fish meat aroma (**B**), cured meat aroma (**C**), and fried seafood aroma (**D**). The positive half-axis in the figure indicates positive contribution, the negative half-axis indicates negative contribution, and the striped columns indicate that the X variables have significant contributions to the Y variables (*p* < 0.05).

To further investigate the contribution of each volatile substance to individual aroma sensory attributes, PLS1 contribution analysis was performed. Figure 6 showed the results of the PLS1 contribution analysis, which describes the contributions of volatile substances to the aroma sensory attributes: fried seafood aroma, fish meat aroma, braised beef in soy sauce aroma, and cured meat aroma. In the figure, the positive half-axis indicates a positive contribution, while the negative half-axis indicates a negative contribution. Striped bars represent X variables that have a significant contribution to y variables (*p* < 0.05). As shown in the figure, nonanal had a significant negative contribution to braised beef in soy sauce aroma. All volatile substances explained 59% of the variation in the braised beef in soy sauce aroma. In the sensory evaluation (Figure 2), sample 5c was found to have a higher score for braised beef in soy sauce aroma, while in the volatile compound analysis (Figure 4A), this sample had a lower OAV for nonanal, which was consistent with the result that nonanal has a negative contribution to braised beef in soy sauce aroma. Dibutyl sulfide, eucalyptol, and 2-acetylpyrazine had significant positive contributions to fish meat aroma. All volatile substances explained 46% of the variation in the fish meat aroma. In the sensory evaluation, sample 9c had the most prominent fish meat aroma (Figure 2), and this sample also had higher OAVs for dibutyl sulfide and eucalyptol (Figure 4A). Additionally, these three substances are mainly derived from spices, and their positive contribution to fish meat aroma confirmed the synergistic effect between spice flavors and the inherent aroma of fish meat. (1R)-(+)-α-pinene and sabinene had significant positive and negative contributions to cured meat aroma, respectively. All volatile substances explained 62% of the variation in the cured meat aroma. As mentioned in Section 3.3, (1R)-(+)-α-pinene has a fresh pine wood aroma, which may further impart rancid, smoky, and roasted flavors to fried foods through Maillard reactions or lipid oxidation [36]. By combining OAVs and sensory evaluation scores, it was observed that some samples (such as 1a, 4b, 9c, and 10d) had both high OAVs of (1R)-(+)-α-pinene and high scores for cured meat aroma. It was indicated that the aroma of (1R)-(+)-α-pinene was highly matched the sensory characteristics of cured meat aroma, thus showing a positive contribution. Benzaldehyde, eucalyptol, and 2-acetylpyrazine had significant negative contributions to fried seafood aroma. All volatile substances explained 68% of the variation in the fried seafood aroma. Fried seafood aroma is a composite aroma of fat oxidation flavor from high-temperature frying and the inherent flavor of seafood. However, the almond aroma of benzaldehyde, the mint aroma of eucalyptol, and the smoky aroma of 2-acetylpyrazine may conflict with this composite aroma, resulting in negative contributions. Furthermore, among the samples (5c-9c) with adjusted chili oil formulations, the OAVs of eucalyptol and 2-acetylpyrazine were lower than those of commercial products, while their sensory scores for fried seafood aroma were higher than those of commercial products. This phenomenon further confirmed the analysis result that both substances have negative contributions to fried seafood aroma.

#### 2.4.2. Predictability of Aroma Sensory Properties Based on Key Flavor Compounds

The above results confirmed that there was a certain correlation between volatile substances and aroma sensory properties. Therefore, in order to realize the rapid prediction of the impact of volatile substances on sensory aroma, a prediction model of volatile substances on aroma sensory attributes was established. Additionally, the credibility and accuracy of the prediction model were evaluated. To investigate the predictability of aroma sensory intensity from volatile substance data, the test samples were projected onto the calibration models optimized through PLSR cross-validation, which were built based on key volatile substances (X matrix) and aroma sensory scores (Y matrix). The calibration models—C-model braised beef in soy sauce, C-model fish meat, C-model cured meat and C-model fried seafood—were based on data from known samples. The test samples were predicted according to these calibration models.

**Figure 7:**
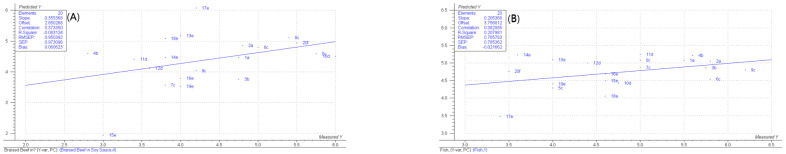

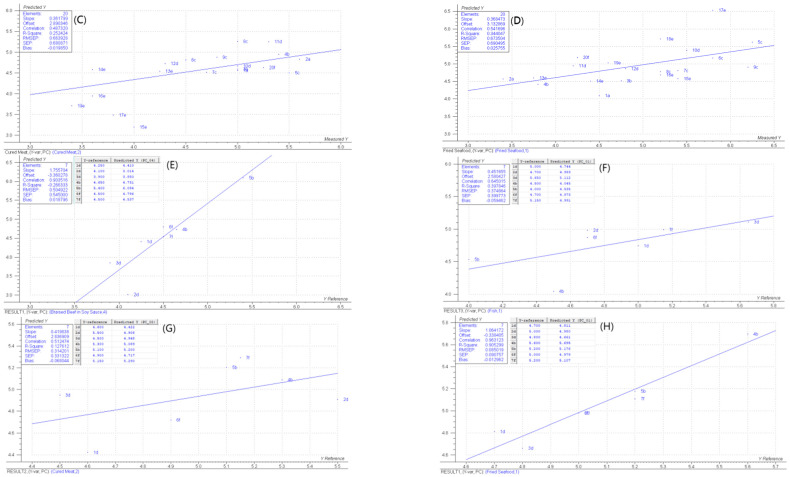
Prediction of aroma sensory scores based on key volatile compound data. The numbers represent sample codes, and different letters indicate individual modifications of different processes based on the commercial spicy anchovy products (**A**–**H**). (**A**) Prediction model for braised beef in soy sauce aroma; (**B**) Prediction model for fish meat aroma; (**C**) Prediction model for cured meat aroma; (**D**) Prediction model for fried seafood aroma; (**E**) Validation model for braised beef in soy sauce aroma; (**F**) Validation model for fish meat aroma; (**G**) Validation model for cured meat aroma; (**H**) Validation model for fried seafood aroma.

Studies on the validation models showed that the explained variance of braised beef in soy sauce aroma increased from 1 to 4 PCs. Therefore, an optimized model involving 4 PCs was selected for prediction. Similar judgments were made for the remaining three sensory attributes, and optimized models with 1, 2, and 1 PCs were established, respectively. Figure 7A–D show the validation models obtained from cross-validation. On this basis, the correlation coefficients and root mean square error of prediction (RMSEP) were calculated. The results showed that the correlation coefficients of the four validation models were not very high, with the highest being for predicting fried seafood aroma (r = 0.542). However, it was found that the accuracy of the four validation models was relatively good (RMSEP = 0.95, 0.77, 0.66, and 0.67; the smaller the error, the better), and the shifts in the data from the calibration models were small (offsets = 2.85, 3.75, 2.89, 3.13). The phenomenon that sample positions were distributed far on both sides of the trend line indicated that the predicted values were higher or lower than the measured values. For example, sample 17e in Figure 7A, located above the trend line, indicated a higher predicted value (approximately 1.23 times the actual measured value), while sample 15e, located below the trend line, indicated a lower predicted value (0.76 times the actual measured value). In conclusion, the validation models have a certain degree of predictability for these four aromas.

Based on the quantitative prediction model established above, the key volatile substance data of the test samples were projected into the model to predict their aroma sensory properties. The results are shown in Figure 7E–H. The correlation coefficients of braised beef in soy sauce aroma (r = 0.90) and fried seafood aroma (r = 0.96) are relatively high, indicating that these two models can better predict the changes in aroma sensory properties of these 7 samples. For braised beef in soy sauce aroma, in addition to the fact that nonanal has a significant negative contribution to it from the PLS1 contribution analysis, benzaldehyde and 2-acetylpyrazine identified in volatile compounds may be the core substances with positive contributions. This is because both are typical products of the Maillard reaction during the sauce-making process. They highly match the salty and mellow characteristics of braised beef in soy sauce aroma, and their content changes may indirectly and synergistically enhance the richness of the sauce and halogen flavor. Since nonanal mainly comes from the high-temperature oxidation of fatty acids. Reducing the frying temperature may reduce the formation of nonanal. While prolonging the frying time to retain more benzaldehyde through a mild Maillard reaction. This can not only strengthen the braised beef in soy sauce aroma but also avoid it being masked by the over-oxidized oil aroma. For fried seafood aroma, although it is known from the PLS1 analysis that benzaldehyde, eucalyptol, and 2-acetylpyrazine have significant negative contributions to fried seafood aroma. Combined with the observation of sensory evaluation and changes in substance OAVs, it can be found that olefin compounds such as (1R)-(+)-α-pinene and (E)-β-ocimene were potential positive contributors to fried seafood aroma. Because their contents are generally increased in samples with high scores for fried seafood aroma. These substances have a fatty and herbal aroma, which can synergize with aldehydes produced by lipid oxidation during frying to strengthen the characteristic frying flavor of seafood. To further regulate the content of these substances, on the one hand, high-temperature frying (180–200 °C) can be used to accelerate lipid oxidation and Maillard reaction in fish meat, increasing the content of olefin substances and strengthening the frying base aroma. On the other hand, the frying time can be moderately controlled to reduce the production of phenylalanine degradation products (benzaldehyde) and excessive Maillard reaction products (2-acetylpyrazine), avoiding their interference with fried seafood aroma. In addition, from the result that the reduction in the content of negative contributing substances (eucalyptol and 2-acetylpyrazine) found in samples 5c-9c with modified chili oil formulations has a positive effect on improving fried seafood aroma. It is known that the proportion of spices in the chili oil formulation can also be optimized, such as reducing the amount of fennel and star anise. This may also be a key process path to reduce the content of such substances. The correlation coefficients of fish meat aroma (r = 0.64) and cured meat aroma (r = 0.51) were relatively low, indicating that there was a certain error between the actual aroma sensory values and the predicted values of the samples. It can be seen from Figure 5 that fish meat aroma and cured meat aroma are affected by many substances. The correlation coefficient of the validation model itself was only about 0.5. Therefore, the poor prediction effect was also due to their insufficient correlation. Sensory evaluators may more easily perceive braised beef in soy sauce aroma and fried seafood aroma clearly. Thus, the prediction effect was relatively satisfactory. In addition, chili is involved in the preparation process of spicy anchovy products. Existing studies have shown that the intake of capsaicin can affect people’s perception of flavor [53]. Therefore, the relationship between volatile substances and aroma sensory properties may be more complex. However, it can be clearly proved from the model data that test samples with independent key volatile substance datasets can be successfully projected onto the calibration model for aroma sensory prediction.

## 3. Materials and Methods

### 3.1. Materials

Anchovies with body length of 5.00 ± 1.00 cm and a mass of 3.85 ± 0.65 g were caught in the South China Sea. After being quick-frozen, they were transported to Pingjiang Jinzai Food Co., Ltd. (Changsha, China) via cold chain at −20 ± 1 °C. The test samples included commercial spicy anchovy products and 20 types of finished spicy anchovy products with different processing parameters and significant differences in sensory quality, which were obtained by making single changes to the key processes involved in the commercial products. All samples were prepared, vacuum-packaged, and sterilized at Jinzai Food Co., Ltd. (Yueyang, China), then mailed to the laboratory of Shanghai Institute of Technology and stored in a ventilated and cool place. The modified processes or formulas for each sample are shown in Table 2. Due to commercial confidentiality, the specific methods of process changes are not disclosed. The internal standards 1,2-dichlorobenzene, dichloromethane (analytical reagent, AR), and anhydrous sodium sulfate (analytical reagent, AR) were obtained from Titan Scientific Co., Ltd. (Shanghai, China). The n-alkane mixtures, consisting of C_7_–C_30_ straight-chain alkanes, were from Aladdin Chem (Shanghai, China). The reference aroma compounds for identification and quantitation experiments were ≥95% pure for GC, purchased from Aladdin Chem (Shanghai, China).

### 3.2. The Basic Processing Flow of Spicy Anchovies

The raw materials were soaked and cleaned thoroughly before being placed in an oil pan for frying. They are then transferred to brine and continuously stirred for marination. Subsequently, seasonings such as chili oil, powder, and paste were added and mixed. The product was divided into bags. Afterwards, the bags were sealed and sterilized.

### 3.3. Sensory Evaluation and Construction of Flavor Wheel

Sensory characteristic description was conducted by a 30-member sensory evaluation panel from Shanghai Institute of Technology (with a male-to-female ratio of 1:1 and ages ranging from 20 to 28). This panel was selected from 40 candidates in accordance with ISO 8586:2023 standards [54], covering the processes of selection, training, and supervision. The evaluators were required to receive a total of 8 h training, which was carried out in four sessions (2 h each, completed within two weeks).

In accordance with ISO 8589-2007 standards, descriptive terms were collected for 21 types of spicy anchovy samples in the sensory laboratory of Shanghai Institute of Technology. With reference to the research methods of previous flavor wheels [25,55,56], members of the sensory evaluation panel were required to conduct spontaneous descriptive analysis on the overall characteristics (including appearance, aroma, etc.) of all spicy anchovy samples. No communication between members was allowed during this period. All descriptive terms were collected. Hedonic terms (such as “delicious” and “tasty”), quantitative terms (such as “a bit salty” and “too spicy”), and vague terms (such as “strange taste” and “bad smell”) were removed. Repeated descriptions and terms with similar meanings were merged. Only terms that accurately refer to a certain smell and have a mention rate over 5% were kept [13]. Thus, 136 sensory descriptive terms were obtained, including 56 aroma descriptors, 38 taste descriptors, 23 taste feel descriptors, 14 spiciness descriptors, and 5 appearance descriptors (Table S1). The flavor wheel for spicy anchovies was composed of these descriptive terms. Through group discussions, a total of 17 descriptive terms and their corresponding standard reference substances were determined (Table 2). The sensory panel was trained with the reference substances corresponding to each sensory descriptor for a total of 6 h over two weeks, enabling the panel members to be familiar with each sensory attribute and to reach a consensus: oily sheen (freshly fried fish), brown (chocolate), fried seafood aroma (fried seafood), fish meat aroma (steamed sea bass), braised beef in soy sauce aroma (traditional braised beef in soy sauce), cured meat aroma (dried cured fish), sweetness (sucrose aqueous solution), umami (monosodium glutamate), fishy off-flavor (fresh sea fish), saltiness (edible salt), braised sauce taste (marinated duck products), chewy (beef jerky), juicy (roast meat skewers), tough (cooked chicken breast), initial spiciness (fire noodle seasoning), delayed spiciness (Sichuan hot pot base), and strong aftertaste spiciness (Sichuan hot pot base) (Table 3).

Subsequently, the sensory panel was asked to evaluate each descriptor of each sample using a 9-point scale, where “1–3” indicates weak intensity, “4–6” indicates moderate intensity, and “7–9” indicates strong intensity. The average score of each descriptor was taken as the intensity value of that descriptor, thereby determining the intensity values of each sensory attribute for the 21 spicy anchovy samples. This study was declared to the Ethics Committee of Shanghai Institute of Technology (Shanghai, China). Written informed consent was obtained from the participants or their legal guardians. The spicy anchovy samples caused no foreseeable risk or discomfort to the participants.

### 3.4. Extraction of Volatile Flavor Compounds

#### 3.4.1. SAFE

The fishes were put into a meat grinder (Zhejiang Shaoxing Supor Small Appliance Co., Ltd., Shaoxing, China) and crushed into minced meat. Exactly 30.0 g of the minced meat was weighed, to which 400 mL of dichloromethane and 300 μL of internal standard o-dichlorobenzene were added. The mixture was sealed and magnetically stirred at room temperature for 3 h. The solution was collected by filtration, and the filtrate was extracted using a SAFE device (Beijing Kangbaite Technology Co., Ltd., Beijing, China) at 40 °C for 2 h under a pressure of 5 × 10^−5^ Pa. After the sample in the collection bottle was thawed, anhydrous sodium sulfate was added for drying to remove water for more than 2 h, followed by filtration. The filtrate was concentrated to 10 mL by rotary evaporation and further concentrated to 1 mL using nitrogen blowing. The final extract was stored at −20 °C until GC-MS analysis.

#### 3.4.2. SPME

First, 0.3 g of minced fish meat was weighed into a clean and odorless headspace vial (15 mL). Then, 3 mL of saturated saline solution was added and mixed well prior to addition of 10 μL of o-dichlorobenzene (100 ppm) as the internal standard. The headspace vial was sealed with a silicone septum and subjected to GC-MS analysis.

### 3.5. GC-MS

Chromatographic conditions: Qualitative analysis was conducted using HP-INNOWAX (60 m × 0.25 mm × 0.25 μm) and DB-5 (60 m × 0.25 mm × 0.25 μm) capillary columns (Agilent, Santa Clara, CA, USA). The inlet temperature was 250 °C, the injection volume was 2 μL, and the splitless mode was adopted. Temperature program: The column temperature was maintained at 40 °C, then increased to 100 °C at 8 °C/min and held for 3 min, followed by increase to 175 °C at 12 °C/min and held for 5 min, and finally increased to 220 °C at 10 °C/min and held for 8 min. The carrier gas was nitrogen with a flow rate of 1 mL/min.

Mass spectrometric conditions: The electron ionization (EI) energy was 70 eV, the ion source temperature was set at 230 °C, and the mass scanning range was 45–400 *m*/*z* in full scan mode.

### 3.6. Qualitative and Quantitative Analysis of Volatile Compounds

Qualitative analysis: The mass spectra of unknown compounds were compared with the NIST11.L spectral library for qualitative identification. Under the same instrumental conditions, n-alkanes C7-C30 were injected, and the retention indices (RI) were calculated based on the peak emergence times of the n-alkanes and volatile compounds for qualitative verification by comparing with references. The volatiles were identified in terms of mass spectra, retention indices (RI) with reference values, standard compounds of the identified odorants, and odor descriptions of authentic standards. The reference is from National Institute of Standards and Technology (NIST) 11 and database: https://webbook.nist.gov/ [19].

Quantification: The internal standard method was used for quantification. The amount of each compound was calculated by comparison with a certain amount of the internal standard substance o-dichlorobenzene at a concentration of 100 ppm.

### 3.7. OAV

Odor activity value (OAV) is calculated by dividing the concentration of an odor substance by the respective orthonasal odor threshold. Compounds with an OAV ≥ 1 are the characteristic aroma substances in the sample; the larger the OAV, the greater the contribution of the substance to the aroma of the sample.

### 3.8. AEDA

The key aroma components in commercial spicy anchovy products were analyzed using the AEDA method combined with GC-O via FD factors [18]. The concentrated extracts were diluted with dichloromethane in gradients of 1:2, 1:4, 1:8, …, 1:1024. Each diluted solution was subjected to sensory evaluation by GC-O until no odor could be detected. Each panel member conducted the analysis three times.

### 3.9. AECA

The AECA method is an analytical approach with an operational process opposite to that of the AEDA method. Based on the previous studies with slight modifications [57], the extract (approximately 200 mL) after treatment with the SAFE device was directly subjected to sniffing and recording via GC-O without concentration. Subsequently, the extract was gradually concentrated to 160 mL, 80 mL, 40 mL, 20 mL, 10 mL, 5 mL, and 2.5 mL. Sniffing and recording were performed at each concentration level. The maximum extract volume at which a compound could be detected by smell was recorded. A larger extract volume indicates a greater contribution of the substance to the overall odor of the sample. Each panel member conducted the analysis three times.

### 3.10. GC-O

For commercial spicy anchovy products, GC-O analysis was conducted by combining the AEDA method and AECA method to identify the aroma-active compounds. The GC-O analysis equipment used was an Agilent 7890 GC (Santa Clara, CA, USA) equipped with an FID and an olfactory detection port (ODP 2). The effluent was split between the FID and the sniffing port at a ratio of 1:1, and the temperature program was the same as that used for GC-MS.

### 3.11. Statistical Analysis

SIMCA 14.1 software was used to process sensory data. Microsoft Excel 2010 was employed for statistical data analysis. Origin 2022 was utilized to draw flavor wheels and radar charts. Unscrambler version 9.7 was applied for PLSR analysis and model construction.

## 4. Conclusions

This study focused on the effects of changes in processing parameters and formulations on the sensory attributes and aroma composition of spicy anchovies. A flavor wheel covering 5 modalities and 17 key sensory descriptors was built via QDA. As can be seen from the sensory evaluation, due to the capsaicin in Xiaomila chili peppers, the score of Sample 5c for “initial spiciness” reached 6.8/9, and the “fishy off-flavor” of Sample 9c was reduced to 2.1/9 via linalyl acetate in Sichuan pepper. SPME-GC-MS showed olefins were the most abundant volatile compounds, accounting for 46.6% of all identified volatiles, with (1R)-(+)-α-pinene (OAV = 38.3–84.12) and (E)-β-ocimene (OAV = 9.42–17.96) having OAV > 1 in all samples and positively correlating with “fried seafood aroma” (r = 0.96). Integration of OAVs and sensory evaluation data further elucidated the regulatory role of core processing steps in flavor formation. Specifically, the “fried seafood aroma” of spicy anchovies intensified with the accumulation of olefins ((1R)-(+)-α-pinene, (E)-β-ocimene), which was promoted by lipase pretreatment of fish embryos via accelerated lipid hydrolysis and oxidation. This aroma was also synergistically strengthened by aldehydes (e.g., nonanal) and olefins generated when frying at 180 °C, a temperature that balanced lipid oxidation and Maillard reactions. By comparison, 200 °C frying caused overproduction of burnt substances (e.g., excessive 2-acetylpyrazine), masking anchovies’ inherent “fish meat aroma” and impairing flavor. A PLSR model, built by correlating key volatiles (OAV > 1) with sensory attributes, achieved high prediction accuracy for “braised beef in soy sauce aroma” (r = 0.90 with RMSEP = 0.66, respectively) and “fried seafood aroma” (r = 0.96 with RMSEP = 0.67, respectively). This study provides support for spicy anchovy process optimization and quality control, with future research to explore aroma substance synergies for directional flavor regulation.

## Figures and Tables

**Table 1:** AEDA and AECA of aroma active compounds in the commercial spicy anchovy products.

Compound	Odorant Description	FD Factor ^1^	Volume/mL ^2^	Identification ^3^
Olefins				
D-limonene	lemon	32	80	MS, RI, S, O
terpinene	turpentine, woody, herbal	2	80	MS, RI, S, O
cis-anethole	fennel, pungent, herbal	4	40	MS, RI, S, O
sabinene	pine wood	- ^4^	2.5	MS, RI, S, O
γ-terpinene	pine wood, herbal	-	2.5	MS, RI, S, O
α-curcumene	pungent, herbal	-	2.5	MS, RI, S, O
zingiberene	ginger, woody	-	2.5	MS, RI, S, O
myrcene	sweet, cinnamon, woody	2	40	MS, RI, S, O
2-carene	green, herbal	-	2.5	MS, RI, S, O
terpinolene	pine wood, herbal	4	10	MS, RI, S, O
Alcohols				
(-)-4-terpineol	herbal, citrus	-	2.5	MS, RI, S, O
2-methyl-5-(1-methylethyl)-bicyclo[3.1.0] hexan-2-ol	-	-	2.5	MS, O
linalool	camphor	4	-	MS, RI, S, O
diacetone alcohol	alcoholic, slight fruity, sweet	1024	>160	MS, RI, S, O
4-terpineol	pine wood, green, floral	4	10	MS, RI, S, O
3-furanmethanol	green, fruity, floral	4	10	MS, RI, S, O
furfuryl alcohol	irritating odor, bitter, burnt	2	-	MS, RI, S, O
Esters				
linalyl acetate	camphor	4	10	MS, RI, S, O
octaethylene glycol monododecyl ether	floral, fruity, sweet	16	80	MS, RI, S, O
triethyl phosphate	-	-	20	MS, RI, S, O
Phenols				
2-methoxy-5-(prop-2-enyl) phenol	woody, sweet, herbal	4	10	MS, RI, S, O
ethyl maltol	caramel, malt, sweet	8	20	MS, RI, S, O
Others				
4-methyl-5-beta-hydroxyethylthiazole	herbal	2	-	MS, RI, S, O
anethole	fennel, herbal, sweet	32	>160	MS, RI, S, O
2-acetylpyrazine	slight nutty, smoky	4	80	MS, RI, S, O
cis-2-(2-Pentenyl)furan	-	-	2.5	MS, O
2-acetylpyrrole	smoky, bitter	-	2.5	MS, RI, S, O
4-allylanisole	herbal, floral, sweet	4	10	MS, RI, S, O

^1^ FD factors, flavor dilution factor determined on a HP-Innowax column. ^2^ The maximum volume of the solution that can be identified by GC-O olfactory detection within the concentration gradients set in this experiment (>160 mL, 160 mL, 80 mL, 40 mL, 20 mL, 10 mL, 5 mL, 2.5 mL). ^3^ Identification method: MS means identification by comparison with the NIST 11 mass spectra database; RI means confirmed by comparison retention index to reference standards (https://webbook.nist.gov/ (accessed on 5 October 2025)); S means confirmed by authentic standards; O means confirmed by aroma descriptor. ^4^ Means not being detected.

**Table 2:** Table of samples with different processes or formulations.

Sample	The Modified Process						
	Additive-Treated Fish Embryos ^1^	Frying Process ^2^	Chili Oil Recipe ^3^	Preparation Method of Chili Oil ^4^	Seasoning Paste ^5^	Seasoning Powder ^5^	Specific Parameters
1a	√						lipases
2a	√						proteases
3b		√					180 °C
4b		√					200 °C
5c			√				Xiaomila chili pepper
6c			√				Zidantou chili pepper
7c			√				Erjingtiao chili pepper
8c			√				a low proportion of Sichuan pepper
9c			√				a high proportion of Sichuan pepper
10d				√			oil frying
11d				√			oil pouring
12d				√			oil soaking
13e					√		beef paste
14e					√		chicken paste
15e					√		Beef:chicken = 1:1.5
16e					√		Beef:chicken = 1.5:1
17e					√		Beef:chicken = 1:2
18e					√		Beef:chicken = 2:1
19e					√		no paste ^6^
20f						√	chicken powder:beef powder = 1:1

^1^ Lipase and proteases were used as additives. ^2^ Frying was conducted at different temperatures. ^3^ Differences between samples lay in the types or contents of chili peppers, Sichuan peppercorns, and capsaicin. ^4^ The Chili oil was prepared by frying, oil-pouring, and oil-soaking. ^5^ Differences between samples were attributed to variations in the types and proportions of beef paste/powder and chicken paste/powder. ^6^ It referred to the complete absence of any protein-based paste (including beef paste and chicken paste), and only basic seasonings (salt, sugar, spices, etc.) were retained in the seasoning system.

**Table 3:** Final vocabulary of the descriptive sensory analysis of spicy anchovies.

Sensory Attributes	Descriptors	Reference Substances
Appearance	Oily sheen	Freshly fried fish
	Brown	Chocolate
Aroma	Fried seafood	Fried seafood
	Fish meat	Steamed sea bass
	Braised beef in soy sauce	Traditional braised beef in soy sauce
	Cured meat	Air-dried cured fish
Taste	Sweetness	Aqueous sucrose solution
	Umami	Monosodium glutamate
	Fishy off-flavor	Fresh sea fish
	Saltiness	Edible salt
	Braised sauce	Marinated duck products
Taste feel	Chewiness	Beef jerky
	Juiciness	Grilled meat skewers
	Toughness	Cooked chicken breast
Spiciness	Initial spiciness	Spicy instant noodle seasoning
	Delayed spiciness	Sichuan hotpot base
	Strong aftertaste	Sichuan hotpot base

## Data Availability

Other experimental data are available in the Supplementary Materials. Additional data are available upon request to authors.

## Supplementary Materials

The following supporting information can be downloaded at: https://www.mdpi.com/article/10.3390/molecules31010057/s1, Table S1: A list of 136 descriptive terms, including 56 aroma (orthonasal olfaction) descriptors, 43 taste (retronasal olfaction) descriptors, 18 taste feel descriptors, 14 spiciness descriptors, and 5 appearance descriptors; Table S2: Geometric mean (M) and variance of sensory attributes of spicy anchovies; Table S3: Odor Activity Values (OAVs) of Volatile Compounds in Spicy Anchovies; Figure S1: DModX (left) and Hotelling’s T2 test (right) of the characteristic descriptors of spicy anchovies; Figure S2: Principal Component Analysis (PCA) and Hierarchical Clustering Analysis (HCA) of the characteristic descriptors of spicy anchovies.
