# Supplementary material for: Process-Modulated Flavor Formation and Establishment of Predictive Modeling for Aroma in Spicy Anchovies
Source: Molecules. 2025 Dec 23;31(1):57. doi: 10.3390/molecules31010057 (PMC12786851; doi:10.3390/molecules31010057)
Supplement: Supplementary file 1 [file molecules-31-00057-s001.zip › molecules-3996771-supplementary.pdf]

molecules

Supplementary materials

Process-modulated Flavor Formation and Establishment of Predictive Modeling for Aroma in Spicy Anchovies

Zishan Liao[1], Qian Liu[1], Wenli Kang[2,3], Zemin Ding[2,3], Shixian Yin[2,3] and Shiqing Song[1,*]

[1] Faculty of Flavour Fragrance and Cosmetics,Shanghai Institute of Technology, Shanghai 201418, China
[2] Jinzai Food Group Co.,Ltd., Yueyang, Hunan 410400, China
[3] Pingjiang Jinzai Food Co.,Ltd., Yueyang, Hunan 410400, China
* Author to whom correspondence should be addressed.

Abstract

Research on spicy anchovies lacks dedicated sensory frameworks, reliable aroma identification, and systematic processing–flavor insights. In this study, 21 spicy anchovy samples with different processing parameters were selected as research objects. The effects of process modifications on the sensory attributes and aroma composition of spicy anchovies were investigated through sensory evaluation and aroma analysis. A product-specific flavor wheel (5 modalities, 136 terms) with 17 key descriptors was built via Quantitative Descriptive Analysis. GC-O combined with AEDA/AECA identified 13 key aroma compounds in the commercial sample. HS-SPME-GC-MS detected 73 volatiles across all samples, among which olefins (34 species) were dominant and their formation was linked to lipid oxidation and high-temperature processing. Odor activity values and sensory data revealed that a frying temperature of 180 °C promoted nonanal and (E)-β-ocimene to enhance "fried seafood aroma"; Xiaomila chili pepper boosted "initial spiciness" via capsaicin; and high Sichuan pepper masked "fishy off-flavor" via linalyl acetate. A prediction model for aroma sensory attributes was established and the prediction correlations for "braised beef in soy sauce aroma" and "fried seafood aroma" were relatively high (r = 0.90 and 0.96, respectively). This study provides theoretical guidance for the flavor improvement of spicy anchovies.

Keywords: Spicy anchovies; flavor wheel; Gas chromatography-mass spectrometry/olfactometry; Partial least squares regression analysis; Prediction model

Academic Editor: Francesco Cacciola

Received: 4 November 2025
Revised: 11 December 2025
Accepted: 19 December 2025
Published: 23 December 2025

Copyright: © 2025 by the authors. Licensee MDPI, Basel, Switzerland. This article is an open access article distributed under the terms and conditions of the Creative Commons Attribution (CC BY) license.

Table S1. A list of 136 descriptive terms, including 56 aroma (orthonasal olfaction) descriptors, 43 taste (retronasal olfaction) descriptors, 18 taste feel descriptors, 14 spiciness descriptors, and 5 appearance descriptors.

Main sensory modalities	Secondary terms	Descriptors
	Meaty	Fish
	Meaty	Beef
	Meaty	Sausage
	Meaty	Squid
	Meaty	Mutton
	Meaty	Dried meat floss
	Meaty	Meat dumplings
	Meaty	Cured meat
	Sweet	Honey
	Sweet	Teriyaki
	Sweet	Fresh-sweet
	Sweet	Herb
	Pungency	Chili powder
	Pungency	Sharp pungency
	Lipid	Greasy off-flavor
	Lipid	Spicy oil
	Lipid	Sesame oil
	Lipid	Scallion oil
	Umami	Seafood
Orthonasal olfaction	Umami	Monosodium glutamate
	Umami	Fermented soybean
	Salty	Sea salt
	Salty	Pickled vegetable
	Salty	Salt meat
	Salty	Spiced salt
	Salty	Kelp
	Bean	Tofu
	Bean	Dried tofu
	Burnt	Grilled fish
	Burnt	Fried fish skin
	Burnt	Teppanyaki
	Burnt	Smoky
	Burnt	Barbecue
	Condiment	Nori
	Condiment	Sesame
	Condiment	Cumin
	Condiment	Sichuan pepper
	Condiment	Pickled potherb mustard
	Condiment	Mushroom

	Condiment	Pickled cabbage
	Condiment	Allium
	Condiment	Ginger
	Fishy	Fishy off-flavor
	Fishy	Putrid fishy
	Fishy	Earthy
	Special odor	Sweat
	Special odor	Sour fermented
	Special odor	Cat food
	Special odor	Wood
	Special odor	Pickled
	Special odor	Plastic
	Special odor	Saliva
	Special odor	Musty
	Special odor	Preserved mustard greens
	Special odor	Banana
	Special odor	Rice
	Meaty	Fish
	Meaty	Dried shrimp
	Meaty	Pork
	Meaty	Squid
	Meaty	Cured meat
	Meaty	Beef jerk
	Fishy	Fishy off-flavor
	Fishy	Sea fishy
	Sweetness	Bake caramelized sweetness
	Oily	Lard
	Oily	Scallion oil
	Condiment	Pepper
	Condiment	Garlic
Retronasal olfaction	Condiment	Ginger
	Condiment	Five-spice
	Condiment	Sesame
	Condiment	Sichuan pepper
	Soy product	Tofu
	Soy product	Fermented tofu
	Soy product	Dried beancurd stick
	Soy product	Spiced dried tofu
	Fruitwood	Nutty
	Fermentation	Rancid oil
	Fermentation	Alcohol
	Fermentation	Pickled product
	Fermentation	Fermented black bean
	Fermentation	Preserved mustard greens

	Burnt	Scorched
	Burnt	Pancake
	Burnt	Barbecue
	Special odor	Nori
	Special odor	Rubber
	Special odor	Mushroom
	Special odor	Crispy noodle snack
	Special odor	Gasoline
	Special odor	Saliva
	Special odor	Earthy
	Special odor	Astringency
Taste feel	Basic taste	Sourness
	Basic taste	Sweetness
	Basic taste	Bitterness
	Basic taste	Saltiness
	Basic taste	Umami
	Juiciness	Shrivelled
	Juiciness	Juicy
	Juiciness	Greasy
	Juiciness	After-sweetness
	Chewiness	Firm
	Chewiness	Thick
	Chewiness	Brittle
	Chewiness	Chewy
	Chewiness	Crispness
	Texture	Soft
	Texture	Hard
	Texture	Fluffy
	Texture	Glutinous
	Texture	Tender
	Texture	Tough
	Texture	Flaky
	Texture	Gritty
	Texture	Smooth
Spiciness	Sensation	Sweet and spicy
	Sensation	Fragrant and spicy
	Sensation	Dry spicy
	Sensation	Throat-burning spicy
	Sensation	Numbing-spicy
	Degree	Mild
	Degree	Medium
	Degree	Extreme
	Complexity	Initial
	Complexity	Delayed

	Complexity	Long-lasting
	Complexity	Strong aftereffect
	Complexity	Mellow
	Complexity	Sharp
	Color	Caramel
	Color	Reddish brown
Appearance	Color	Dark brown
	Luster	Oily sheen
	Luster	Dull grayish

Table S2. Geometric mean (M) and variance of sensory attributes of spicy anchovies.

Descriptors	F(%)[1]	I(%)[2]	M(%)[3]
**Appearance**			
Oily sheen	100.0	63.3	79.6
Dull grayish	92.9	42.4	62.7
Caramel	88.1	46.2	63.8
Reddish brown	85.7	42.9	60.6
Dark brown	78.6	38.1	54.7
**Orthonasal olfaction**			
Fish	100.0	57.1	75.6
Beef	78.6	25.2	44.5
Sausage	31.0	11.9	19.2
Squid	83.3	37.6	56.0
Meat dumplings	40.5	15.2	24.8
Cured meat	59.5	20.0	34.5
Honey	26.2	8.6	15.0
Teriyaki	59.5	20.5	34.9
Chili powder	85.7	36.7	56.1
Greasy off-flavor	64.3	27.6	42.1
Sesame oil	90.5	41.4	61.2
Scallion oil	31.0	8.6	16.3
Monosodium glutamate	66.7	31.4	45.8
Fermented soybean	83.3	38.1	56.3
Spiced salt	47.6	18.6	29.7
Kelp	14.3	3.8	7.4
Tofu	35.7	8.6	17.5
Dried tofu	47.6	20.5	31.2
Grilled fish	90.5	41.9	61.6
Fried fish skin	81.0	36.7	54.5
Smoky	83.3	37.1	55.6
Barbecue	78.6	37.6	54.4
Sesame	21.4	6.7	12.0

Cumin	69.0	26.2	42.5
Sichuan pepper	40.5	11.4	21.5
Mushroom	42.9	16.2	26.3
Pickled cabbage	23.8	7.1	13.0
Allium	21.4	6.7	12.0
Ginger	9.5	2.4	4.8
Fishy off-flavor	83.3	41.9	59.1
Putrid fishy	57.1	25.2	38.0
Earthy	16.7	4.3	8.5
Sour fermented	26.2	11.0	16.9
Wood	9.5	2.4	4.8
Plastic	16.7	4.3	8.5
Saliva	21.4	7.1	12.4
Musty	19.0	4.3	9.0
Preserved mustard greens	38.1	15.2	24.1
Rice	7.1	1.9	3.7
**Retronasal olfaction**			
Fishy off-flavor	71.4	41.9	54.7
Fish	97.6	59.0	75.9
Dried shrimp	54.8	19.0	32.3
Pork	31.0	10.0	17.6
Squid	78.6	29.5	48.2
Cured meat	40.5	12.9	22.8
Beef jerk	50.0	19.0	30.9
Bake caramelized sweetness	14.3	4.3	7.8
Lard	35.7	15.7	23.7
Scallion oil	26.2	8.1	14.6
Pepper	38.1	12.9	22.1
Garlic	23.8	11.0	16.1
Ginger	19.0	5.2	10.0
Five-spice	78.6	37.6	54.4
Sesame	40.5	11.0	21.1
Sichuan pepper	54.8	18.1	31.5
Tofu	35.7	10.0	18.9
Fermented tofu	33.3	10.0	18.3
Nutty	2.4	0.5	1.1
Rancid oil	31.0	10.0	17.6
Alcohol	21.4	7.1	12.4
Pickled product	57.1	27.6	39.7
Fermented black bean	54.8	29.5	40.2
Preserved mustard greens	35.7	13.3	21.8
Scorched	31.0	12.9	19.9
Barbecue	78.6	32.4	50.4
Rubber	16.7	3.8	8.0

Mushroom	31.0	9.0	16.7
Earthy	26.2	10.5	16.6
**Taste feel**			
Sourness	28.6	8.6	15.6
Sweetness	85.7	32.4	52.7
Bitterness	47.6	19.5	30.5
Saltiness	95.2	50.5	69.3
Umami	81.0	41.4	57.9
Shrivelled	76.2	30.5	48.2
Juicy	95.2	46.2	66.3
Greasy	81.0	34.8	53.0
After-sweetness	54.8	20.5	33.5
Soft	66.7	30.0	44.7
Hard	76.2	36.7	52.9
Firm	85.7	44.3	61.6
Thick	78.6	38.1	54.7
Chewy	85.7	44.3	61.6
Fluffy	64.3	22.9	38.3
Glutinous	42.9	15.2	25.6
Tender	50.0	15.7	28.0
Tough	76.2	34.8	51.5
Gritty	59.5	22.9	36.9
Smooth	40.5	12.4	22.4
**Spiciness**			
Initial	95.2	52.4	70.6
Delayed	97.6	46.2	67.1
Dry spicy	81.0	31.0	50.1
Sweet and spicy	64.3	24.8	39.9
Numbing-spicy	64.3	28.1	42.5
Throat-burning spicy	61.9	27.1	41.0
Extreme	38.1	11.0	20.4
Mild	78.6	27.6	46.6
Strong aftereffect	85.7	41.0	59.2
Fragrant and spicy	81.0	39.5	56.6
Long-lasting	83.3	41.0	58.4
Medium	71.4	32.9	48.4
Mellow	50.0	18.1	30.1
Sharp	59.5	25.2	38.8

[1] The percentage of the actual number of times a descriptive term is mentioned relative to the total number of times that descriptive term could potentially be mentioned.

[2] The percentage of the intensity score of a descriptive term actually given by the evaluation panel relative to the maximum possible intensity score for that descriptive term.

[3] $M = \sqrt{F \times I}$

Table S3. Odor Activity Values (OAVs) of Volatile Compounds in Spicy Anchovies.

Compound	Odor threshold (μg/kg)	OAV										
		0	1a	2a	3b	4b	5c	6c	7c	8c	9c	10d
Dibutyl sulfide	0.00088	1386.04	790.42	705.21	711.46	758.25	396.74	648.71	884.71	649.47	751.69	625.36
2-Nonanone	0.041	2.49	2.56	2.47	2.62	3.04	2.61	2.87	2.54	2.69	2.57	2.64
(+)-Carvone	0.16	1.07	1.05	0.71	0.78	0.84	1.01	0.89	0.79	0.64	0.88	0.75
Benzaldehyde	0.75089	1.48	1.24	1.29	1.17	1.32	1.16	1.18	1.18	1.21	1.18	1.19
Nonanal	0.0011	268.47	216.36	282.57	276.74	368.38	231.36	264.9	350.34	298.97	319.91	261.72
Geranyl acetate	0.15	3.26	3.89	3.24	3.78	3.48	2.68	2.94	2.9	3.24	3.39	2.92
(1R)-(+)-α-Pinene	0.0022	38.3	66.27	65.45	74.64	84.12	66.76	64.51	46.65	81.59	77.39	54.19
(E)-β-Ocimene	0.034	12.42	14.01	11.99	16.62	15.52	11.95	11.6	11.21	10.63	9.55	9.42
Sabinene	0.98	1.25	0.49	0.49	0.62	0.74	0.51	0.35	0.47	0.4	0.59	0.6
β-Phellandrene	0.5	1.23	0.92	0	2.13	2.21	0.83	0.81	0	0.86	0.91	0.83
Eucalyptol	1.1	2.24	1.41	1.3	1.14	1.22	0.85	1.04	1.09	1.05	0.95	0.89
Anethole	0.057	24.46	50.88	32.61	33.02	51.43	12.86	14.06	12.96	12.66	12.73	12.41
2-Acetylpyrazine	0.06	7.93	7.64	7.18	6.37	6.15	4.86	6.47	7.17	6.75	7.01	5.67

Table S3.(continued)

Compound	Odor threshold (μg/kg)	OAV									
		11d	12d	13e	14e	15e	16e	17e	18e	19e	20f
Dibutyl sulfide	0.00088	740.26	668	772.25	757.75	741.32	844.36	475.83	440.89	592.59	624.28
2-Nonanone	0.041	2.45	2.94	2.58	2.48	2.63	3.13	2.96	2.59	2.78	2.84
(+)-Carvone	0.16	0.83	0.71	0.69	0.83	0.81	0.91	1.05	1.01	0.88	0.89

Benzaldehyde	0.75089	1.23	1.19	1.25	1.22	1.17	1.18	0	1.18	1.21	1.21
Nonanal	0.0011	338.42	371.5	277.8	297.15	301.98	284.04	192.08	205.95	229.4	221.96
Geranyl acetate	0.15	3	3.43	3.52	4.34	4.18	4.41	2.97	2.76	4.02	3.36
(1R)-(+)-α-Pinene	0.0022	79.28	59.61	47.65	64.93	49.56	54.23	41.54	59.73	51.95	65.12
(E)-β-Ocimene	0.034	11.36	12.58	14	15.2	17.96	16.25	12.31	11.36	15	11.94
Sabinene	0.98	0.44	0.46	0.63	0.52	1.42	0.55	0.82	0.95	1.28	0.45
β-Phellandrene	0.5	0.93	0.98	1.1	0.99	1.29	1	0.72	0.7	0	0.8
Eucalyptol	1.1	1.01	1.02	1.33	1.12	1.09	1.15	0.79	0.8	1.15	0.95
Anethole	0.057	16.61	15.36	15.73	22.16	23.48	13.37	13.85	23.92	20.48	31.58
2-Acetylpyrazine	0.06	6.78	7.14	6.81	7.23	6.34	6.96	4.55	5.49	6.45	6.15

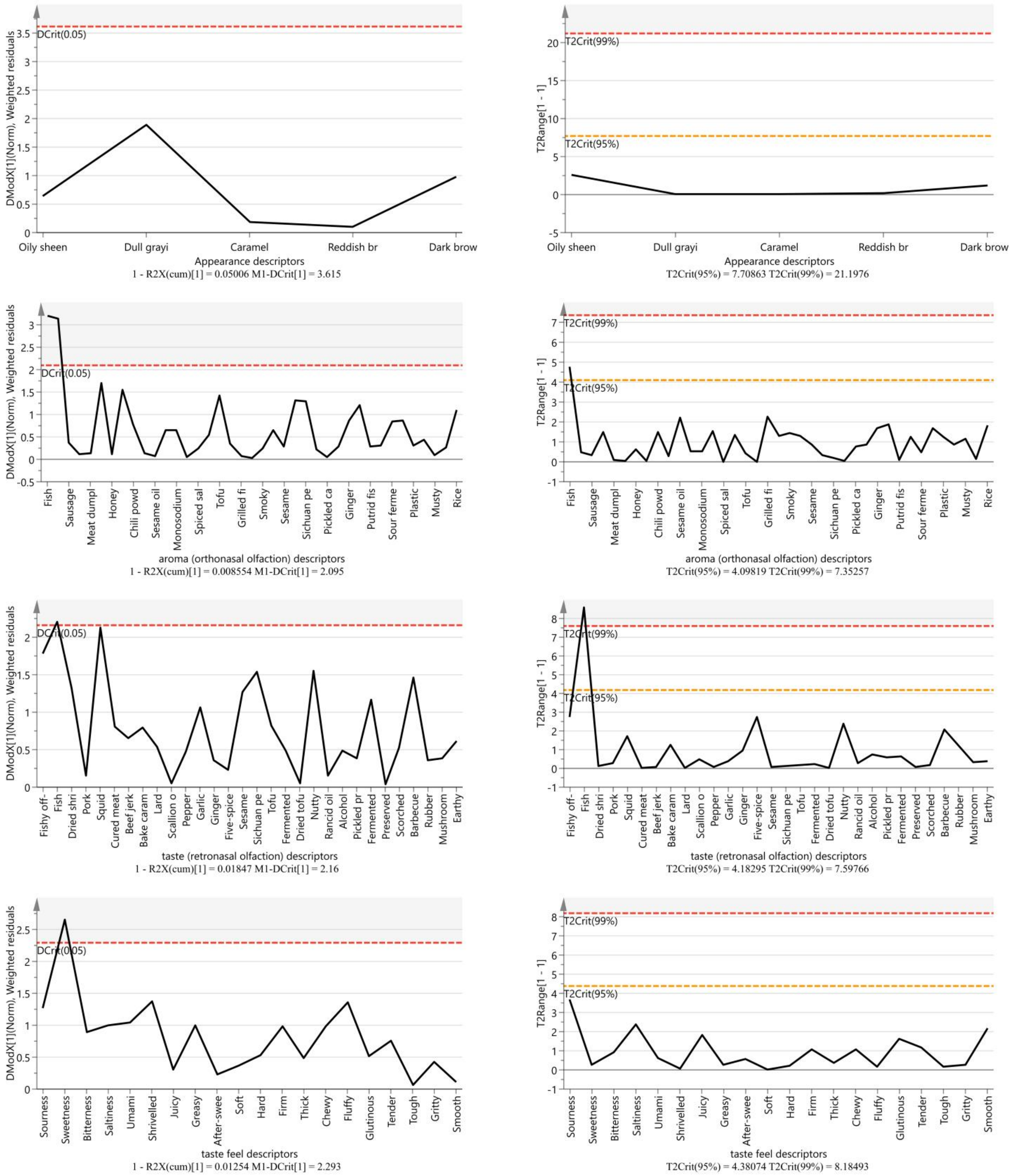

 https://doi.org/10.3390/molecules31010057

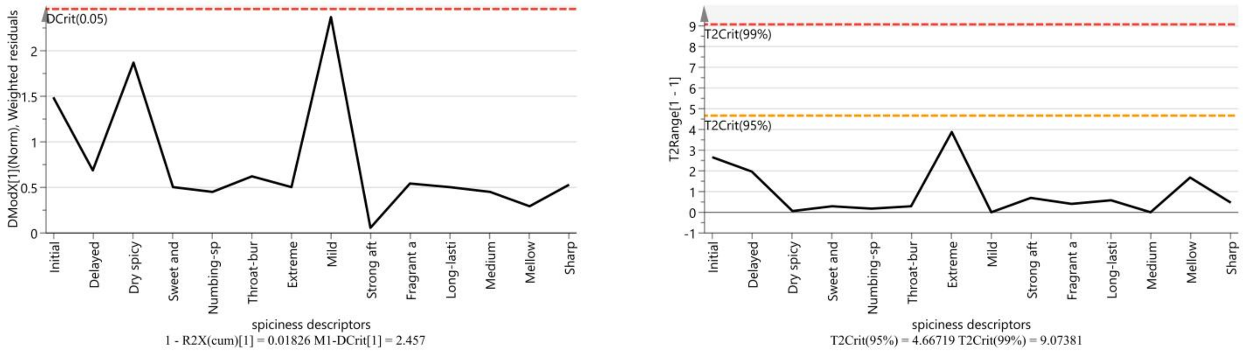

Figure S1. DModX (left) and Hotelling ' s T2 test (right) of the characteristic descriptors of spicy anchovies.

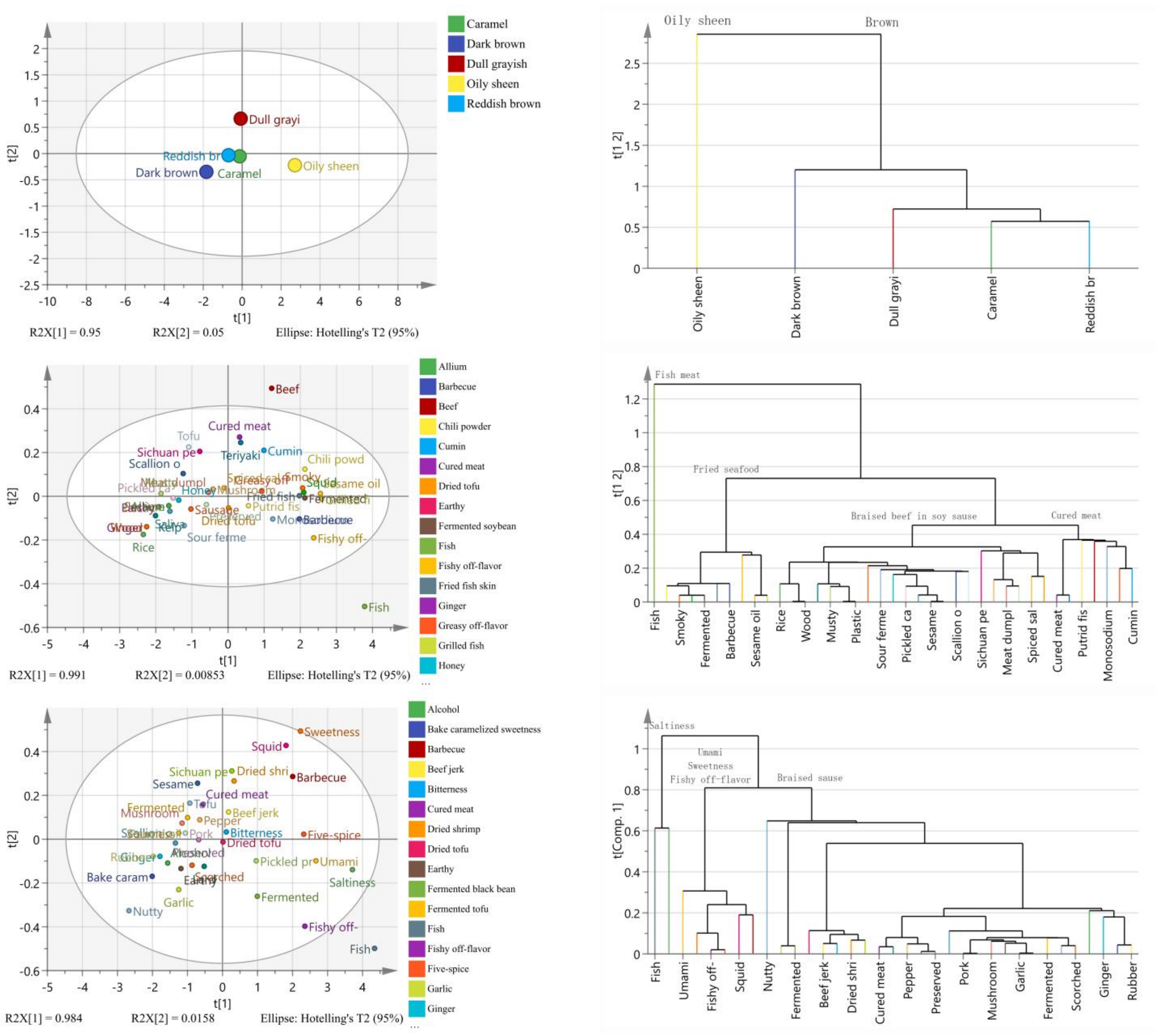

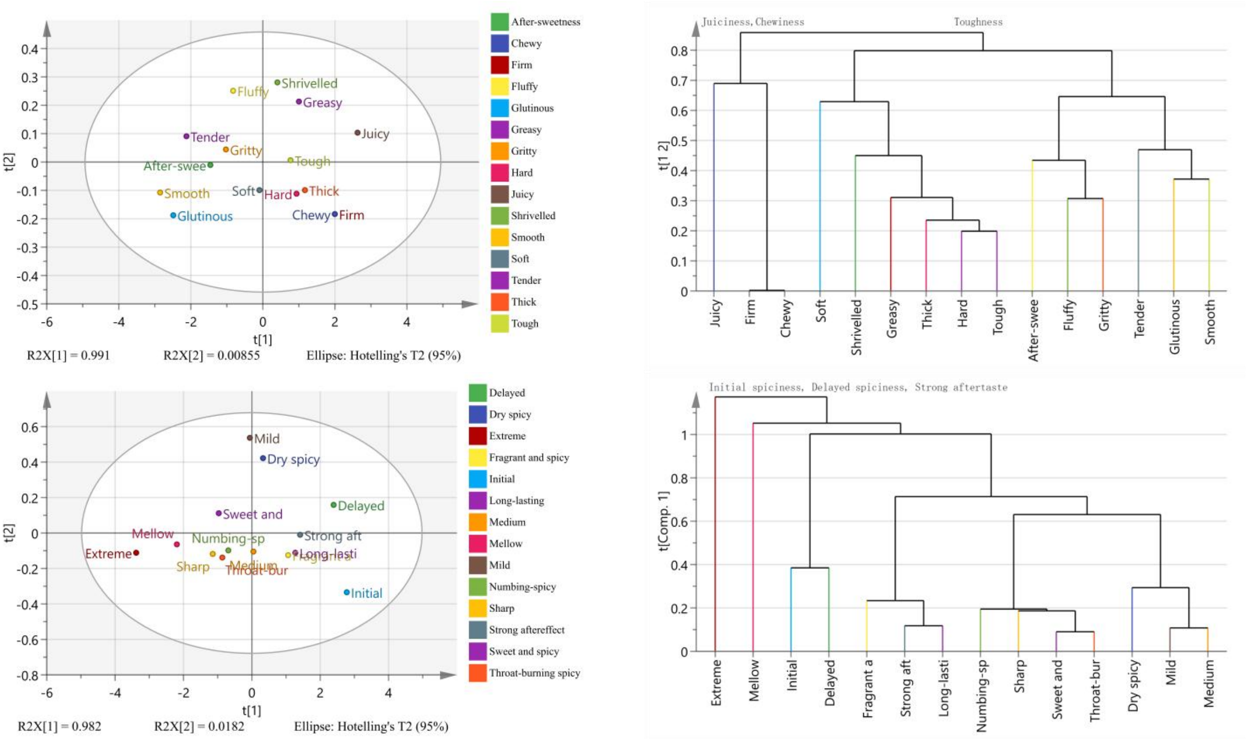

Figure S2. Principal Component Analysis (PCA) and Hierarchical Clustering Analysis (HCA) of the characteristic descriptors of spicy anchovies.
